# Metabolic-epigenetic regulation of the HBV cccDNA reservoir: pathways underpinning viral persistence

**DOI:** 10.3389/fmicb.2026.1968789

**Published:** 2026-10-12

**Authors:** Qing Peng, Liyuan Hao, Shenghao Li, Fei Yu, Na Li, Fanghang Ye, Xiaoyu Hu

**Affiliations:** 1 Clinical Medical College, Chengdu University of Traditional Chinese Medicine, Chengdu, Sichuan, China; 2 Department of Infectious Diseases, Hospital of Chengdu University of Traditional Chinese Medicine, Chengdu, Sichuan, China; 3 School of Integrated Traditional Chinese and Western Medicine, Hebei University of Chinese Medicine, Shijiazhuang, Hebei, China; 4 Department of Integrated Traditional Chinese and Western Medicine Oncology, The Fourth Hospital of Hebei Medical University, Shijiazhuang, Hebei, China

**Keywords:** cccDNA, functional cure, hepatitis B virus, immune tolerance, metabolic-epigenetic regulation

## Abstract

Chronic hepatitis B virus (HBV) infection remains a global public health burden, and functional cure represents the primary goal of clinical management. The nuclear covalently closed circular DNA (cccDNA) minichromosome stably maintained in infected hepatocytes constitutes the major barrier to HBV cure. Clinically, functional cure is defined as sustained hepatitis B surface antigen (HBsAg) seroclearance with undetectable serum HBV DNA after finite treatment, a definition that does not require complete cccDNA elimination. At the molecular level, functional cure is thought to be associated with stable transcriptional silencing of cccDNA and integrated HBV DNA, a state biologically distinct from physical cccDNA depletion. Accumulating data demonstrate that the persistence and transcriptional homeostasis of the cccDNA reservoir are controlled by viral proteins and dynamically shaped by host metabolic signaling and epigenetic remodeling. Here, we first outline key steps of the HBV life cycle to establish the mechanistic context for cccDNA formation and replenishment, and then focus our synthesis on the integrated host metabolic-epigenetic network that governs cccDNA establishment, transcriptional activity, stability, and clearance. We dissect how central carbon metabolites, bile acid signaling, histone post-translational modifications, and immune cytokines converge to modulate cccDNA reservoir homeostasis, and critically evaluate the strength of evidence supporting their physiological relevance in the chronically infected human liver. We also discuss mechanisms of HBV immune evasion, including hepatitis B virus X protein (HBx)-driven epigenetic reprogramming and subviral particle-associated immune tolerance. We summarize preclinical and clinical therapeutic strategies targeting the metabolic-epigenetic microenvironment, viral assembly, antigen secretion, and cccDNA modulation to achieve a functional cure for HBV infection. Finally, we outline key unresolved challenges in the field, including the undefined *in vivo* cccDNA turnover kinetics, the poorly characterized physiological relevance of cell-to-cell transmission, the inherent limitations of gene-editing technologies, and the urgent need for specific non-invasive biomarkers reflecting intrahepatic cccDNA activity.

## 1 Introduction

Hepatitis B virus (HBV) infection remains a major global public health challenge and the seventh leading cause of infectious disease-related death worldwide, driving liver cirrhosis, hepatocellular carcinoma (HCC), and other virus-related liver complications (Danpanichkul et al., 2025). According to the latest estimates from the World Health Organization (WHO), hepatitis B surface antigen (HBsAg) seroprevalence is approximately 3.2%, with 254 million individuals living with chronic infection and an estimated 820,000 annual deaths attributed to HBV-related disorders (Polaris Observatory Collaborators, 2023). China bears a substantial burden of HBV, with approximately 86 million affected individuals, over one-third of all chronic infections worldwide (Hui et al., 2024). Notably, genotype C, which predominates in China, accounting for approximately 60% of chronic infections (Chan et al., 2004), is associated with a higher risk of HCC than other genotypes (Choi et al., 2023; Feitelson et al., 2022; Lu et al., 2021; Rawat and Bouchard, 2015). Transmission occurs primarily via perinatal exposure and contact with infected bodily fluids (Rudramurthy et al., 2024). Whereas most adults achieve spontaneous viral clearance, more than 90% of neonates infected perinatally develop chronic infection and face long-term risks of developing cirrhosis and HCC (Ren C. et al., 2024).

A highly effective prophylactic vaccine is available, offering a favorable safety profile and durable protection, particularly when administered during infancy or at birth (Romano’ and Zanetti, 2022). At present, nucleos(t)ide analogs (NAs) and interferons (IFNs) constitute the main therapeutic options for chronic HBV infection (Wang J. et al., 2020). Whereas current therapies primarily aim to prevent disease progression to cirrhosis or liver cancer, NAs effectively suppress HBV replication but require lifelong administration, while pegylated interferon (Peg-IFN)-α offers curative potential for some patients but is limited by side effects (Boonstra and Sari, 2025). The nuclear covalently closed circular DNA (cccDNA) minichromosome constitutes a stable intrahepatic viral reservoir and its persistence represents the main barrier to HBV cure, since currently approved therapies are unable to eliminate cccDNA (Ren et al., 2025). Complete HBV cure is defined as the total eradication of HBV to allow safe treatment cessation without virological relapse or liver disease progression, yet it is clinically unattainable due to persistent intrahepatic cccDNA and integrated HBV DNA in the host genome, both of which can cause viral reactivation even in recovered patients (Lok et al., 2017). Clinically, HBV functional cure is defined as sustained HBsAg seroclearance with undetectable serum HBV DNA after finite antiviral treatment (Ghany et al., 2023). This definition relies entirely on peripheral serological and virological markers and does not require elimination or even reduction of intrahepatic cccDNA, as cccDNA cannot be routinely quantified in clinical settings (Ghany et al., 2023). At the molecular level, functional cure is presumed to be closely associated with stable transcriptional silencing of intrahepatic cccDNA and integrated HBV DNA, a state biologically distinct from partial or complete physical depletion of the cccDNA reservoir (Kumar et al., 2024).

To overcome these clinical limitations, it is imperative to elucidate the molecular mechanisms underlying HBV persistence. Here, we review the key molecular pathways governing the HBV life cycle, with a focus on the homeostatic regulation of the cccDNA reservoir. We dissect how each stage of viral infection contributes to establishing, amplifying, or silencing the cccDNA minichromosome, and highlight the interplay between viral proteins, host metabolism, epigenetic remodeling, and immune surveillance that sustains this persistent viral reservoir. We further critically assess the translational relevance of these pathways, highlighting where preclinical evidence remains to be validated in the chronically infected human liver. By integrating insights spanning viral entry, virion egress, and immune evasion, we identify emerging therapeutic targets capable of disrupting cccDNA homeostasis to facilitate curative strategies for chronic HBV infection.

## 2 Search strategy and study selection

Literature retrieval was conducted in PubMed, web of Science, and Scopus, covering publications from 1980 to 2026, with the final search performed in august 2026. The search strings combined the following terms including “hepatitis B virus,” “cccDNA,” “metabolism,” “epigenetic regulation,” “hepatocyte,” “liver fibrosis,” and “functional cure” using boolean operators (AND/OR). Peer-reviewed English original research papers and authoritative review articles were included. Case reports, conference abstracts, and non-peer-reviewed gray literature were excluded. Duplicate records were manually removed. Publications were screened based on their relevance to the core research question, namely the identification of metabolic and epigenetic mediators that control cccDNA persistence in chronically infected hepatocytes. Landmark studies describing the HBV life cycle, viral entry, and DNA integration were manually supplemented to establish the mechanistic context.

## 3 HBV virology and viral structure

A detailed understanding of HBV virology, genome organization, and the molecular basis of cccDNA formation is a prerequisite for interpreting how host metabolic and epigenetic pathways shape viral persistence. HBV is a small enveloped hepadnavirus that selectively infects hepatocytes and induces acute and chronic liver injury, cirrhosis, and HCC. It possesses a 3.2-kilobase (kb) partially double-stranded relaxed-circular DNA (rcDNA) genome, consisting of a full-length negative strand covalently bound to viral polymerase (Pol) and an incomplete positive strand (Gerlich and Robinson, 1980). The HBV genome contains four overlapping open reading frames (C, P, S, and X) that encode, respectively, the core structural proteins hepatitis B core antigen (HBcAg) and hepatitis B e antigen (HBeAg), viral DNA Pol, envelope proteins HBsAg, and the regulatory hepatitis B virus X protein (HBx) (Boonstra and Sari, 2025). Among these viral components, HBx is critical for modulating host epigenetic and metabolic signaling to sustain viral persistence. The nuclear cccDNA minichromosome serves as the sole persistent transcriptional template for all viral RNAs and supports ongoing viral replication and chronic infection (Liang, 2009; Tropberger et al., 2015). Its establishment, transcriptional activity, and stability are dynamically governed by host DNA repair machinery, metabolic cues, and epigenetic remodeling. Due to its nuclear stability and self-sustaining capacity, the cccDNA reservoir constitutes the primary obstacle to HBV functional cure (Figure 1).

**FIGURE 1:**
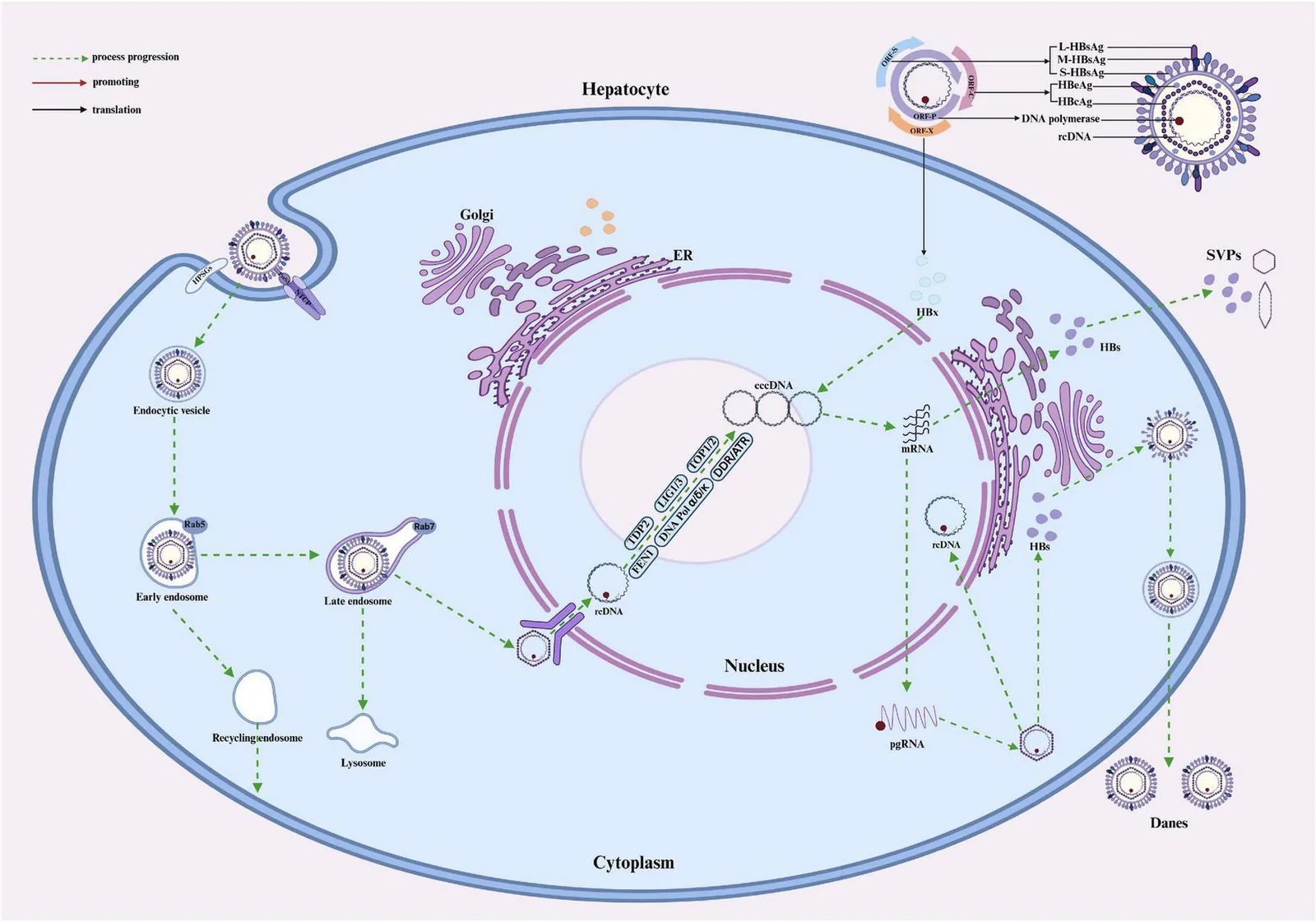
HBV life cycle and cccDNA biogenesis in hepatocytes. Following NTCP-mediated viral entry, nucleocapsids are transported to the nucleus, where rcDNA is repaired to form the cccDNA minichromosome. cccDNA serves as the template for all viral transcripts. Mature nucleocapsids either re-enter the nucleus to amplify the cccDNA pool or acquire envelope proteins for secretion. HSPGs, heparan sulfate proteoglycans; E-cadherin, epithelial cadherin; NTCP, sodium taurocholate co-transporting polypeptide; PreS1, pre-surface 1; Rab5, ras-related protein Rab-5; Rab7, ras-related protein Rab-7; Golgi, Golgi apparatus; ER, endoplasmic reticulum; rcDNA, relaxed-circular DNA; TDP2, tyrosyl-DNA phosphodiesterase 2; FEN1, flap endonuclease 1; LIG1, ligase 1; LIG3: ligase 3; TOP1, topoisomerase 1; TOP2, topoisomerase 2; DNA Pol α, DNA polymerase α; DNA Pol δ, DNA polymerase δ; DNA Pol κ, DNA polymerase κ; DDR, DNA damage response; ATR, ataxia telangiectasia and RAD3-related; cccDNA, covalently closed circular DNA; mRNA, messenger RNA; pgRNA, pregenomic RNA; HBs, hepatitis B surface; SVPs, subviral particles; ORF, open reading frames; L-HBsAg, large hepatitis B surface antigen; M-HBsAg, middle hepatitis B surface antigen; S-HBsAg, small hepatitis B surface antigen; HBeAg, hepatitis B e antigen; HBcAg, hepatitis B core antigen; HBx, hepatitis B virus X protein. (This figure was created in https://BioRender.com).

## 4 Initiation of HBV infection: viral entry and nuclear transport pathways

HBV utilizes hepatocyte-specific sodium taurocholate co-transporting polypeptide (NTCP) as its primary entry receptor (Yan et al., 2012). Infection initiates via low-affinity binding to cell-surface heparan sulfate proteoglycans (HSPGs) (Li and Wands, 2016; Verrier et al., 2016), followed by high-affinity interaction between the myristoylated PreS1 motif of the HBV envelope protein and NTCP, triggering clathrin-dependent virion endocytosis (De Falco et al., 2001; Fukano et al., 2018). After internalization, viral nucleocapsids escape endosomal compartments and are transported along microtubules to the nuclear periphery. At the nuclear pore complex (NPC), mature nucleocapsids undergo regulated uncoating, releasing rcDNA into the nucleoplasm; rcDNA acts as the direct precursor for cccDNA biogenesis (Rabe et al., 2009; Schmitz et al., 2010). Following nuclear delivery of rcDNA, host DNA repair machinery converts the partially double-stranded viral genome into cccDNA. The size and transcriptional competence of this initial cccDNA pool are therefore set at the earliest steps of infection, directly linking viral entry pathways to the host-directed regulation of the reservoir (Figure 1).

## 5 Core of HBV persistence: cccDNA regulatory pathways

### 5.1 Genetic and structural regulation

#### 5.1.1 rcDNA repair mechanisms

cccDNA is indispensable for the HBV life cycle and the maintenance of chronic infection, residing in the nucleus of infected hepatocytes as the master transcriptional template for all viral RNAs, including pregenomic RNA (pgRNA), to drive ongoing synthesis of viral proteins and progeny rcDNA genomes. The conversion of HBV rcDNA to cccDNA begins within the first 16 h post-infection in primary human hepatocytes and hepatoma cell lines (Tu et al., 2020). cccDNA levels remain constant both *in vitro*, in HBcAg-mutant HBV infection systems, and *in vivo*, in quiescent primary human hepatocytes from HBV-infected humanized mouse models, underscoring its self-sustaining nature in non-dividing cells (Allweiss et al., 2018; Tu et al., 2021a). The marked decline in cccDNA levels in actively dividing hepatocytes, seen both *in vivo* and *in vitro*, suggests the absence of a nuclear retention mechanism for equal distribution to daughter cells, resulting in its dilution over successive cell generations (Allweiss et al., 2018; Tu et al., 2022b). To offset this dilution, HBV depends on *de novo* cccDNA synthesis via new infection and on intracellular recycling of rcDNA-containing capsids, thereby ensuring the maintenance of an adequate cccDNA pool in highly proliferating hepatocytes in cell culture and in animal models (Boonstra and Sari, 2025; Martinez et al., 2021). The conversion of relaxed rcDNA into covalently closed cccDNA is a complex, multi-step process that relies on specific host DNA repair pathways to resolve the unusual gaps and protein adducts of the viral genome. However, the precise host repair factors involved are largely identified from cell-line studies and remain incompletely validated in human liver tissues.

Structurally, rcDNA comprises a full-length coding (−) strand, which is covalently linked at its 5’ end to the viral Pol and features a terminal nine-nucleotide redundancy, alongside a partially complete (+) strand with a capped RNA primer attached to its 5’ end (Nassal, 2008; Tsukuda and Watashi, 2020). Generating cccDNA from rcDNA involves completing the partial (+) strand, eliminating Pol from the (−) strand 5’ terminus, removing the RNA primer from the (+) strand 5’ end, and excising the redundant copy in the nine-nucleotide triple-stranded “r” region to facilitate the ligation of the 5’ and 3’ ends of the two DNA strands.

Of these steps, Pol removal is initially attributed to tyrosyl-DNA phosphodiesterase 2 (TDP2) based on *in vitro* and cell culture studies, although recent evidence from cell lines suggests the involvement of as-yet-unidentified enzymes (Cai et al., 2020; Königer et al., 2014). Concurrently, the redundant overlapping region “r” must be cleaved by flap endonuclease 1 (FEN1) to allow the ligation of the DNA termini by host ligases, such as ligase 1 (LIG1) and ligase 3 (LIG3), as demonstrated primarily in cell culture and *in vitro* reconstitution systems (Kitamura et al., 2018; Long et al., 2017). The completion of the incomplete (+) strand appears to involve distinct Pols depending on the cellular context; while DNA Pol κ is implicated in quiescent cells with low deoxyribonucleoside triphosphates (dNTP) pools, DNA Pol δ and DNA Pol α are suggested to facilitate cccDNA synthesis arising from intracellular recycling, as established primarily in hepatoma cell lines (Ogi and Lehmann, 2006; Qi et al., 2016; Tang et al., 2019), highlighting a potential diversity in repair mechanisms between *de novo* infection and intracellular recycling pathways. Furthermore, the DNA damage response (DDR) kinase ataxia telangiectasia and RAD3-related (ATR) plays a regulatory role, likely by sensing the damaged structure of rcDNA and recruiting the necessary repair machinery (Luo et al., 2020), while topoisomerase 1 (TOP1) and topoisomerase 2 (TOP2) have also been implicated in the circularization process, although the evidence implicating them derives primarily from cell culture studies and their exact mechanisms remain debated (Sheraz et al., 2019). Collectively, these host factors orchestrate the precise conversion of rcDNA into the stable cccDNA minichromosome, establishing the genetic template that is subject to further evolutionary pressure and sequence variation (Figure 1).

#### 5.1.2 cccDNA sequence heterogeneity and HBV DNA integration

Contrary to the conventional view of cccDNA as a homogeneous, static molecular reservoir, the intrahepatic cccDNA population exists as a dynamic quasispecies pool with substantial sequence heterogeneity (Harris et al., 2025). Multiple sources drive this genetic diversity. The error-prone viral reverse transcriptase lacks 3’-5’ proofreading exonuclease activity and introduces mutations at high frequency during pgRNA reverse transcription (González et al., 2018). The multi-component host repair machinery responsible for rcDNA circularization tends to generate incompletely processed repair intermediates, introducing low-level sequence variation into nascent cccDNA templates during lesion repair (Wei and Ploss, 2021a). In parallel, persistent host immune selection pressure enriches viral variants with enhanced replication fitness or immune escape properties (Cento et al., 2013; Schwarz et al., 2025).

Naturally occurring mutations within the cccDNA pool directly reshape viral protein expression, replication capacity, and clinical disease trajectories. The G1896A point mutation in the precore (preC) region introduces a premature stop codon that abrogates HBeAg production, giving rise to HBeAg-negative chronic infection while maintaining active viral replication (Li et al., 2024; Zhao et al., 2023). The A1762T/G1764A double mutation in the basal core promoter (BCP) region reduces preC mRNA transcription and consequently HBeAg expression, while generally enhancing pgRNA synthesis and, in most genetic backgrounds, viral replication capacity (Kumar, 2022). Collectively, clinical and epidemiological studies demonstrate that these preC and BCP variants correlate with accelerated liver fibrosis progression and elevated risk of hepatocellular carcinoma, further illustrating how sequence variation in the cccDNA reservoir shapes clinical outcomes.

Beyond sequence diversity in episomal cccDNA, integration of HBV DNA fragments into the cellular genome represents another critical dimension of viral genome heterogeneity, occurring early during acute infection and accumulating progressively with chronic disease duration, as evidenced by longitudinal clinical studies (Tu et al., 2021b). Integration events preferentially localize to sites of host genomic double-strand breaks, a process mediated by host DDR pathways during rcDNA processing (Bill and Summers, 2004). Integrated HBV sequences are typically truncated, rearranged, and incapable of supporting full-length viral replication or producing infectious virions (Tu et al., 2021b).

Although subviral particles (SVPs) themselves lack viral DNA, integrated HBV DNA can drive robust HBsAg expression, accounting for the massive surplus of non-infectious SVPs that far outnumbers infectious virions in chronic infection (Wooddell et al., 2021). This HBsAg excess leads to persistent immune stimulation, driving progressive exhaustion of HBsAg-specific B cells and impairing HBV-specific T cell function, thereby undermining adaptive immune surveillance (Wooddell et al., 2021). Critically, clinical studies of patients on long-term NA therapy have demonstrated that HBsAg derived from integrated templates persists even when episomal cccDNA transcription is reduced, constituting a major barrier to HBsAg seroclearance and functional cure (Grudda et al., 2022; Taddese et al., 2025). Single-cell and multiplex immunofluorescence studies of patient liver tissues have further clarified the contribution of integrated templates to intrahepatic antigen heterogeneity, revealing that in HBeAg-negative patients, hepatocytes that are HBsAg-positive but hepatitis B core antigen (HBcAg)-negative predominate and often form clonal foci, whereas cells positive for both markers are rare (Aggarwal et al., 2023). These patterns are consistent with transcription from integrated HBV DNA driving HBsAg expression and clonal hepatocyte expansion (Peng et al., 2024).

While genetic and sequence-level features define the composition and diversity of the viral reservoir, the transcriptional activity of both episomal cccDNA and integrated viral sequences is not static but is predominantly governed by epigenetic regulation of chromatin state, which is in turn shaped by host metabolic signaling.

### 5.2 Epigenetic and transcriptional regulation of the cccDNA minichromosome

Within the host nucleus, HBV cccDNA does not exist as naked DNA but as a minichromosome that structurally mimics host chromosomes. Specifically, it is organized into nucleosomes through its association with histones (predominantly H3 and H4) and viral proteins such as HBx and hepatitis B core protein (HBc) (Kumar et al., 2024). The chromatin compaction (ranging from relaxed to condensed states) of this minichromosome is primarily governed by histone post-translational modifications (PTMs), which ultimately dictate whether the viral genome undergoes active transcription or transcriptional silencing (Figure 2).

**FIGURE 2:**
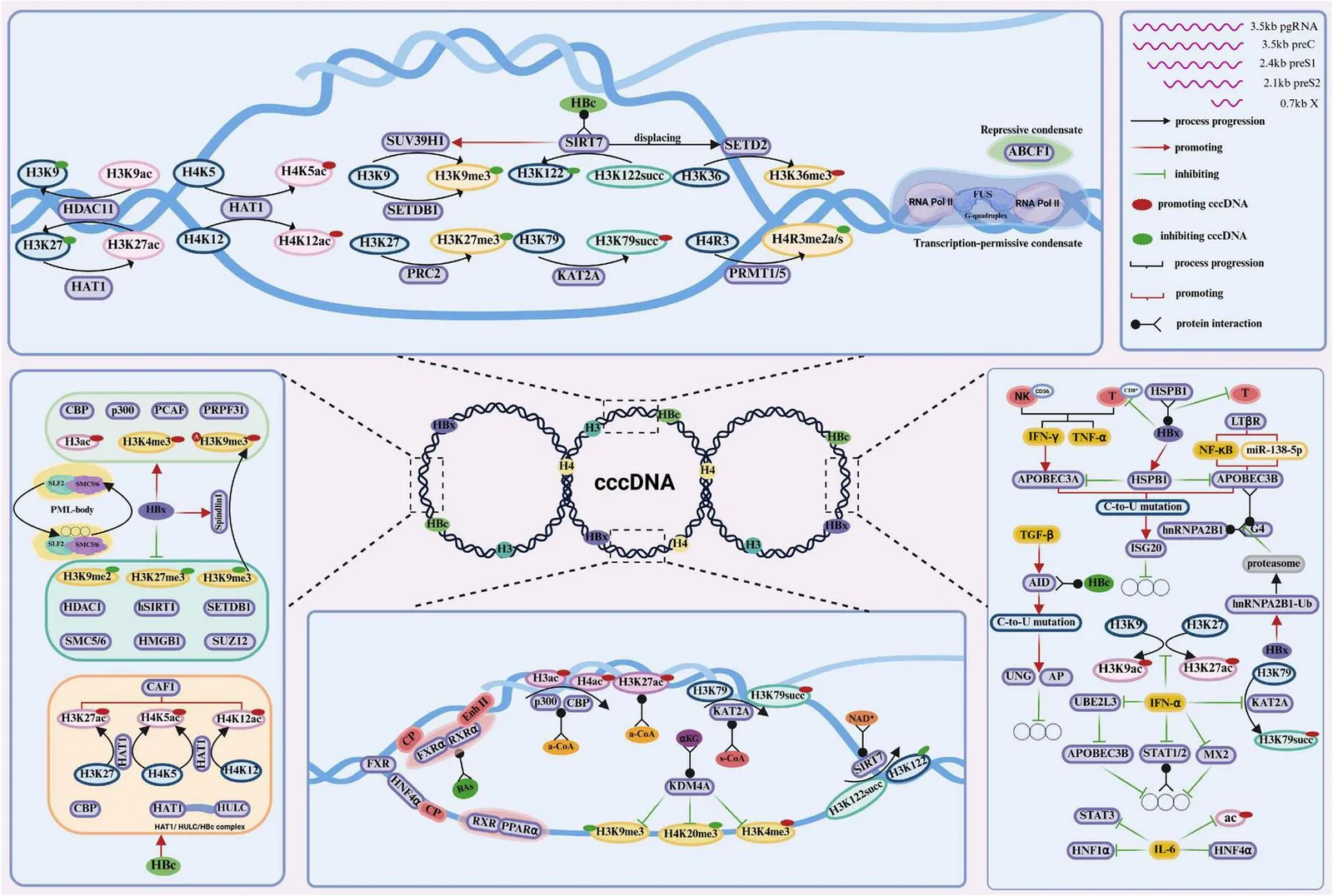
Multi-layered regulatory landscape governing cccDNA homeostasis in HBV-infected hepatocytes. Schematic overview depicting cccDNA organized as a host-like minichromosome. Upper panel illustrates dynamic histone post-translational modifications (acetylation, methylation, succinylation) and liquid-liquid phase-separated condensates that shape cccDNA transcriptional competence. Lower-left sub-panels summarize HBx-driven epigenetic remodeling via PML-body disruption and epigenetic enzyme modulation. Bottom sub-panel highlights metabolite-coupled epigenetic regulation of cccDNA. Right-hand panel shows immune-cytokine-driven host restriction for viral immune evasion. H3K9, histone H3 lysine 9; HDAC11, histone deacetylase 11; H3K9ac, histone H3 lysine 9 acetylation; H3K27, histone H3 lysine 27; HAT1, histone acetyltransferase 1; H3K27ac, histone H3 lysine 27 acetylation; H4K5, histone H4 lysine 5; H4K5ac, histone H4 lysine 5 acetylation; H4K12, histone H4 lysine 12; H4K12ac, histone H4 lysine 12 acetylation; SUV39H1, suppressor of variegation 3-9 homolog 1; H3K9me3, histone H3 lysine 9 trimethylation; SETDB1, SET domain bifurcated histone lysine methyltransferase 1; PRC2, polycomb repressive complex 2; H3K27me3, histone H3 lysine 27 trimethylation; H3K122, histone H3 lysine 122; SIRT7, sirtuin 7; HBc, hepatitis B core protein; H3K122succ, histone H3 lysine 122 succinylation; H3K79, histone H3 lysine 79; KAT2A, lysine acetyltransferase 2A; H3K79succ, histone H3 lysine 79 succinylation; H3K36, histone H3 lysine 36; SETD2, SET domain containing 2, histone lysine methyltransferase; H3K36me3, histone H3 lysine 36 trimethylation; H4R3, histone H4 arginine 3; PRMT5, protein arginine methyltransferase 5; PRMT1, protein arginine methyltransferase 1; H4R3me2s, symmetric dimethylation of histone H4 arginine 3; H4R3me2a, asymmetric dimethylation of histone H4 arginine 3; RNA Pol II, RNA polymerase II; FUS, fused in sarcoma; ABCF1, ATP binding cassette subfamily F member 1; CBP, CREB-binding protein; p300, E1A-binding protein p300; PCAF, p300/CBP-associated factor; PRPF31, pre-mRNA processing factor 31; H3ac, histone H3 acetylation; H3K4me3, histone H3 lysine 4 trimethylation; SMC5, structural maintenance of chromosomes 5; SMC6, structural maintenance of chromosomes 6; SLF2, SMC5-SMC6 complex localization factor 2; PML, promyelocytic leukemia; HBx, hepatitis B virus X protein; H3K9me2, histone H3 lysine 9 dimethylation; HDAC1, histone deacetylase 1; hSIRT1, human sirtuin 1; HMGB1, high mobility group box 1; SUZ12, suppressor of zeste 12 homolog; CAF1, chromatin assembly factor 1; HULC, highly upregulated in liver cancer FXR, farnesoid X receptor; CP, core promoter; FXRα, farnesoid X receptor α; RXRα, retinoid X receptor α; BAs, bile acids; HNF4α, hepatocyte nuclear factor 4α; RXR, retinoid X receptor; PPARα, peroxisome proliferator-activated receptor α;H4ac, histone H4 acetylation; α-CoA, acetyl coenzyme A; KDM4A, lysine demethylase 4A; αKG, α-ketoglutarate; s-CoA, succinyl coenzyme A; NAD^+^, nicotinamide adenine dinucleotide; NK, natural killer; IFN-γ, interferon-γ; TNF-α, tumor necrosis factor α; APOBEC3A, apolipoprotein B mRNA editing enzyme, catalytic polypeptide-like 3A; HSPB1, heat shock protein family B member 1; APOBEC3B, apolipoprotein B mRNA editing enzyme, catalytic polypeptide-like 3B; NF-κB, nuclear factor kappa B; LTβR, lymphotoxin-β receptor; ISG20, interferon-stimulated gene 20; hnRNPA2B1, heterogeneous nuclear ribonucleoprotein A2B1;G4, G-quadruplex; hnRNPA2B1-Ub, heterogeneous nuclear ribonucleoprotein A2B1 polyubiquitination; TGF-β, transforming growth factor -β; UNG, uracil-DNA glycosylase; AP, apurinic/apyrimidinic; HDAC: histone deacetylase; IFN-α, interferon-α; UBE2L3, ubiquitin-conjugating enzyme E2 L3; STAT1, signal transducer and activator of transcription 1; STAT2, signal transducer and activator of transcription 2; MX2, MX dynamin-like GTPase 2; STAT3, signal transducer and activator of transcription 3; HNF1α, hepatocyte nuclear factor-1α; IL-6, interleukin-6; ac, acetylation; pgRNA, pregenomic RNA; preC, cccDNA, covalently closed circular DNA. (This Figure was created in https://BioRender.com).

#### 5.2.1 cccDNA-derived transcription and pgRNA biogenesis

Transcription of the cccDNA minichromosome by host RNA Pol II is a prerequisite for productive HBV replication (Jones and Hu, 2013). The cccDNA genome harbors four overlapping open reading frames, and its transcription is governed by four distinct promoters (C, S1, S2, and X) and two enhancer regions (EnhI and EnhII) (Seeger and Mason, 2015). Among these transcripts, pgRNA, transcribed from the core promoter (CP), is of paramount importance for viral replication (Sun et al., 2014). The CP directs the initiation of two closely related 3.5 kb transcripts: the longer precore mRNA encoding the secreted HBeAg, and the slightly shorter pgRNA (Quarleri, 2014). As a bifunctional viral RNA, pgRNA serves both as the mRNA for core protein and viral Pol translation and as the indispensable template for reverse transcription (Quarleri, 2014). A key structural element governing pgRNA fate is the 5’ epsilon stem-loop, which is specifically recognized by the viral Pol to initiate pgRNA packaging and subsequent reverse transcription within nascent nucleocapsids (Quarleri, 2014). Importantly, studies in infected hepatocyte cell lines have shown that the accessibility of these viral promoters to RNA Pol II and host transcription factors varies with the epigenetic landscape and chromatin compaction state of the cccDNA minichromosome. For instance, in its transcriptionally silent (closed) state, the cccDNA is densely compacted with approximately 15–16 nucleosomes and is marked by repressive histone modifications, which physically hinder transcription factor binding (Nassal, 2015).

#### 5.2.2 Histone modifications and chromatin architecture

Histone acetylation attenuates the electrostatic attraction between histones and DNA, resulting in a relaxed chromatin conformation that facilitates the exposure of DNA sequences to transcription factors. Liver biopsy samples from patients with chronic HBV infection reveal a transcriptionally permissive epigenetic landscape on cccDNA, characterized by abundant activating marks such as histone H3 and histone H4 acetylation and histone H3 lysine 4 (H3K4) trimethylation, but an absence of histone H3 lysine 27 (H3K27) trimethylation and variable levels of histone H3 lysine 9 (H3K9) trimethylation (Flecken et al., 2019; Tropberger et al., 2015). The transcriptional activity of cccDNA is primarily governed by the acetylation status of histones, which is dynamically balanced by the interplay between acetyltransferases and deacetylases. For instance, histone deacetylase 11 (HDAC11) removes acetyl groups from H3K9 and H3K27, thereby inducing epigenetic silencing and suppressing HBV replication in Huh7 cells (Yuan et al., 2019). Conversely, histone acetyltransferase 1 (HAT1) acetylates H3K27, as well as histone H4 lysine 5 (H4K5) and histone H4 lysine 12 (H4K12); this hyperacetylation correlates with enhanced cccDNA transcription and viral propagation across *in vitro*, *in vivo*, and clinical liver tissue studies (Yang et al., 2019). In contrast, *in vitro* and *in vivo* studies have shown that SET domain bifurcated histone lysine methyltransferase 1 (SETDB1) and the polycomb repressive complex 2 (PRC2) mediate H3K9 trimethylation and H3K27 trimethylation, respectively, and drive cccDNA transcriptional silencing (Rivière et al., 2015; Salerno et al., 2020; Zhang et al., 2016). Similarly, protein arginine methyltransferase 5 (PRMT5) and protein arginine methyltransferase 1 (PRMT1) catalyze symmetric and asymmetric dimethylation of histone H4 arginine 3 (H4R3), respectively, and both modifications are linked to repression of cccDNA transcription in cell culture and patient liver tissues (Sun et al., 2022; Zhang et al., 2017). While histone acetylation serves as a primary driver, other PTMs, including those within the globular domain of histones, have also been functionally linked to cccDNA activity. For example, studies in hepatocyte cell lines, primary human hepatocytes, and mouse models demonstrate that histone H3 lysine 79 (H3K79) succinylation promotes transcriptional activation, a modification catalyzed by lysine acetyltransferase 2A (KAT2A) (Qin et al., 2021). Conversely, the nicotinamide adenine dinucleotide (NAD^+^)-dependent desuccinylase sirtuin 7 (SIRT7) is recruited to cccDNA by HBc and mediates histone H3 lysine 122 (H3K122) desuccinylation in cell culture and clinical liver tissues (Yu et al., 2021). This in turn promotes suppressor of variegation 3-9 homolog 1 (SUV39H1)-dependent H3K9 trimethylation deposition and displaces SET domain containing 2, histone lysine methyltransferase (SETD2), thereby reducing histone H3 lysine 36 (H3K36) trimethylation and silencing cccDNA transcription (Yu et al., 2021). Functionally, studies in cell culture and clinical liver tissues demonstrate that active transcription is driven by histone acetylation, whereas the recruitment of histone deacetylase 1 (HDAC1) and the resulting hypoacetylation correlate with reduced viral replication (Pollicino et al., 2006). Furthermore, in cell culture, the histone variant H3.3 has been identified as a positive regulator of HBV gene expression (Alvarez-Astudillo et al., 2021). While metabolic-dependent acylation marks, such as lactylation and crotonylation, have been documented in liver physiology and HCC (Lao et al., 2024), their direct functional roles for cccDNA remain poorly defined.

Beyond covalent histone PTMs, liquid-liquid phase separation (LLPS) has emerged as a novel physical regulatory layer governing cccDNA transcriptional competence. In cell culture models, G-quadruplex secondary structures embedded within cccDNA regulatory regions drive LLPS with fused in sarcoma (FUS), assembling transcription-permissive biomolecular condensates that enrich transcriptional machinery on the viral minichromosome (Giraud et al., 2024b). Conversely, studies in cell culture, animal models, and patient liver tissues have shown that host restriction factors such as ATP-binding cassette subfamily F member 1 (ABCF1) assemble repressive phase-separated condensates with cccDNA, sequestering RNA Pol II and concurrently triggering type I/III interferon responses (Ren C. et al., 2024). Such non-covalent spatial partitioning adds another dimension to cccDNA homeostasis, though evidence in primary human hepatocytes and clinical samples, remains largely lacking.

#### 5.2.3 Regulation by viral proteins

HBV serves as a multifunctional regulator that remodels the cccDNA minichromosome to promote a transcriptionally active state, primarily by modulating the recruitment of chromatin modifiers (Giraud et al., 2024a). In cell culture models, cells expressing an HBx-deficient mutant exhibit transcriptionally silent cccDNA, a state characterized by reduced H3 acetylation and H3K4 trimethylation, elevated levels of repressive marks such as H3K9 dimethylation and H3K9 trimethylation, and enhanced recruitment of HDAC1 and human sirtuin 1 (SIRT1) (Belloni et al., 2009; Rivière et al., 2015). HBx facilitates the recruitment of acetyltransferases, including CREB-binding protein (CBP), E1A-binding protein p300 (p300), and p300/CBP-associated factor (PCAF), to cccDNA, leading to local histone hyperacetylation in cell culture systems (Belloni et al., 2009; Chong et al., 2020; Cougot et al., 2007; Lucifora et al., 2011). Viral transcription is further enhanced through the interaction of HBx with spindlin 1, which shifts the chromatin state from inactive H3K9 trimethylation to active H3K4 trimethylation *in vitro* (Liu et al., 2023). Moreover, HBx disrupts the binding of HBV restriction factors, including the structural maintenance of chromosomes 5 (SMC5)/structural maintenance of chromosomes 6 (SMC6) complex, high mobility group box 1 (HMGB1) and SETDB1, to cccDNA, thereby evading epigenetic silencing in cell culture models (Decorsière et al., 2016; Kim et al., 2022; Rivière et al., 2015). Additionally, HBx interacts with pre-mRNA processing factor 31 (PRPF31) to facilitate its association with the cccDNA minichromosome *in vitro* (Kinoshita et al., 2017). HBx promotes the degradation of suppressor of zeste 12 homolog (SUZ12) within the PRC2 complex, effectively preventing the establishment of H3K27 trimethylation repressive marks in cell culture systems (Zhang et al., 2016). Beyond these indirect modulations, recent findings indicate that proper nucleosome positioning and the chromatin landscape on cccDNA are prerequisites for initiating HBx expression, thereby ensuring viral genome accessibility *in vitro* (Prescott et al., 2025). In turn, HBx directly modulates histone modifications and the chromatin landscape on cccDNA, recruiting histone-modifying enzymes to establish an active viral chromatin state in cell culture models (Alarcon et al., 2016). Beyond canonical epigenetic modulation, the SMC5-SMC6 complex localization factor 2 (SLF2)-SMC5/6 axis restricts cccDNA transcription by sequestering cccDNA in repressive promyelocytic leukemia (PML) bodies; HBx can reverse this spatial compartmentalization, as shown in cell culture models including primary human hepatocytes (Yao et al., 2023).

While HBx is recognized as the predominant regulator of cccDNA activity, emerging evidence has revealed a potential role for HBc in modulating the chromatin state of cccDNA. HBc associates with acetyltransferases, including CBP, on the HBV cccDNA, thereby establishing a transcriptionally permissive chromatin environment in cell culture models (Chong et al., 2017). HBc recruits the histone acetyltransferase 1 (HAT1) to cccDNA, where it assembles into a HAT1/highly upregulated in liver cancer (HULC)/HBc complex in cell culture and human liver-chimeric mouse models (Zhang et al., 2021). Specifically, HAT1 catalyzes the acetylation of H4K5 and H4K12, as well as H3K27 on cccDNA, with the histone chaperone chromatin assembly factor 1 (CAF-1) cooperating to deposit histones, ultimately packaging the viral genome into a transcriptionally active minichromosome structure in cell culture and human liver-chimeric mouse models (Yang et al., 2019). In contrast, other studies have demonstrated that HBc does not participate in cccDNA transcription, as neither its overexpression nor its depletion affects HBV RNA levels in cell culture and mouse models (Tu et al., 2021a; Zhong et al., 2022). Although the role of HBc in cccDNA regulation remains controversial, current evidence supports its context-dependent modulatory functions, whereas its non-redundant contribution to viral persistence *in vivo* remains to be fully elucidated.

The histone modification landscape of the cccDNA minichromosome is not static. Rather, it is dynamically shaped by upstream host metabolic signaling and nuclear receptor activity, which together constitute a central regulatory axis governing viral persistence.

### 5.3 Environmental and metabolic regulation

The transcriptional activity and stability of the hepatic cccDNA reservoir are not an intrinsic properties of the viral minichromosome, but are dynamically coupled to the physiological state of the hepatocyte. Liver-specific environmental cues, intracellular metabolic signaling, and paracrine immune stimuli converge to shape cccDNA persistence through three interconnected regulatory layers, comprising liver-enriched nuclear receptors that act as upstream metabolic sensors, intracellular metabolites that serve as cofactors and substrates for histone modifications, and immune cytokines that mediate non-cytolytic cccDNA silencing and degradation. Crucially, these layers do not operate independently. Nuclear receptors reprogram metabolic flux to alter metabolite availability, while metabolites in turn modulate nuclear receptor activity and epigenetic modifier function. Simultaneously, immune cytokines reshape both cccDNA epigenetic landscapes and hepatocyte metabolic states. Together, this integrated network links hepatic metabolic homeostasis directly to the transcriptional output and stability of the viral reservoir (Figure 2).

#### 5.3.1 Liver-enriched nuclear receptors

Hepatocytes abundantly express liver-enriched nuclear receptors, including hepatocyte nuclear factor 4α (HNF4α), retinoid X receptor (RXR), peroxisome proliferator-activated receptor α (PPARα), and farnesoid X receptor (FXR), which act as master transcription factors governing both hepatic physiological metabolism and HBV cccDNA transcription (Mouzannar et al., 2019; Tang and McLachlan, 2001). Functional binding motifs for these nuclear receptors are enriched within the two enhancer regions of the cccDNA minichromosome.

This regulatory layer is of central importance to chronic HBV persistence because it allows the virus to sense and adapt to fluctuations in hepatic physiology, rather than relying on constitutive viral promoter activity. *In vitro* studies demonstrate that HNF4α directly activates the core promoter by binding conserved cis-elements, driving robust pgRNA synthesis and supporting viral replication (Tang and McLachlan, 2001). PPARα forms heterodimers with RXR to bind its cognate response elements in the HBV genome, and activation by peroxisome proliferators consistently enhances viral pgRNA transcription and replication across cell culture and transgenic mouse models (Dubuquoy et al., 2009). In contrast, although FXR acts as a proviral factor under basal conditions, pharmacological FXR agonists such as GW4064 repress cccDNA transcription and reduce pgRNA synthesis in both cell culture and transgenic models (Mouzannar et al., 2019), revealing a bidirectional regulatory node amenable to therapeutic intervention.

Nuclear receptors do not function as isolated transcription factors; instead, they act at the interface between hepatic metabolism and viral transcription, and their effects on cccDNA intersect closely with downstream metabolite-dependent epigenetic pathways. For example, bile acid (BA)-activated FXR signaling modulates cccDNA promoter activity *in vitro* (Ramière et al., 2008), while also reprogramming hepatic lipid and glucose metabolism (Ma et al., 2006). Similarly, PPARα and HNF4α regulate central carbon and lipid metabolism (Chamouton and Latruffe, 2012; Deans et al., 2023). Metabolites derived from these metabolic pathways, such as acetyl-CoA, function as substrates and cofactors for histone modification (Egger et al., 2024). This integration implies that hepatic physiological shifts can be transmitted to the cccDNA minichromosome not only through direct transcriptional control by nuclear receptors but also indirectly via metabolite-driven epigenetic remodeling.

#### 5.3.2 Metabolite-mediated epigenetic and transcriptional modulation

Hepatic small-molecule metabolites function as upstream signaling mediators to modulate cccDNA status either by activating the above nuclear receptor cascades or by directly mediating epigenetic modifications of cccDNA-bound histones. Rather than acting independently, these metabolites form an interconnected network anchored in central carbon metabolism, where fluctuations in one metabolite cascade through multiple epigenetic modification systems to shape the overall chromatin state of the cccDNA minichromosome.

Bile acids (BAs), the canonical endogenous ligands for FXR, accumulate in hepatocytes under cholestatic conditions and enhance viral pgRNA transcription via farnesoid X receptor α (FXRα)-retinoid X receptor α (RXRα) heterodimers acting on HBV enhancer II and CP regulatory regions, as shown in cell culture models (Kim et al., 2010). This pathway is relevant to chronic infection because it couples viral transcriptional activity to the cholestatic and metabolic alterations that accompany progressive liver fibrosis, potentially supporting reservoir maintenance even in the setting of hepatic injury.

Beyond nuclear receptor ligands, central carbon metabolites such as acetyl coenzyme A (acetyl-CoA) serve as universal acetyl donors for histone lysine acetylation (Sun et al., 2025), directly coupling hepatocyte metabolic flux to cccDNA chromatin accessibility. This coupling is biologically critical for HBV persistence because it allows viral transcription to adapt to the host nutritional and metabolic environment, supporting the maintenance of a transcriptionally permissive reservoir in quiescent, non-dividing hepatocytes, which are the predominant cell type harboring cccDNA in chronic infection. Notably, HBV-induced metabolic reprogramming elevates intracellular acetyl-CoA abundance in clinical specimens, animal models, and cell culture, driving selective enrichment of H3K27 acetylation (Li et al., 2026). This epigenetic remodeling is facilitated by the recruitment of histone acetyltransferases (HATs), such as p300 and CBP, to the cccDNA minichromosome in cell culture models, where these enzymes deposit activating acetylation marks on histones H3 and H4, relaxing the compact viral minichromosome structure and potentiating cccDNA transcriptional activity and viral replication in both cell culture and clinical specimens (Jiang et al., 2025; Pollicino et al., 2006).

Beyond core acetyl-CoA-dependent acetylation, additional metabolic intermediates further remodel cccDNA transcriptional activity by modulating diverse histone methylation signatures. Specifically, *in vitro* studies reveal that α-ketoglutarate (αKG) serves as an indispensable cofactor for the αKG-dependent demethylase lysine demethylase 4A (KDM4A), which removes repressive H3K9 trimethylation and H4K20 trimethylation marks, reduces H3K4 trimethylation deposited on the cccDNA minichromosome, and thereby drives robust transcription of cccDNA (Cheng et al., 2025). For histone succinylation, the tricarboxylic acid cycle intermediate succinyl-CoA serves as a key succinyl donor for KAT2A-mediated deposition of activating H3K79 succinylation on the cccDNA minichromosome to promote viral transcription in both cell culture and animal models (Wang et al., 2017; Yuan et al., 2020). Conversely, sirtuin deacylases such as SIRT7 function in an NAD^+^-dependent manner, catalyzing H3K122 desuccinylation on the cccDNA minichromosome to reinforce transcriptional silencing, a mechanism supported by evidence from cell culture and clinical specimen data (Yu et al., 2021).

Taken together with the nuclear receptor axis above, these metabolite-dependent epigenetic pathways function as individual regulatory modules. Within the chronically infected liver, changes in hepatic homeostasis such as cholestasis, steatosis, or inflammatory injury may alter the abundance of BAs, acetyl-CoA, and αKG, each of which can independently promote permissive cccDNA chromatin states. Meanwhile, metabolites including NAD^+^ and succinyl-CoA participate in counteracting histone acylation events that can restrict cccDNA transcription. Although these pathways operate in isolation in most experimental systems, their effects may converge on the cccDNA minichromosome within the same hepatocyte. The magnitude and hierarchy of such convergence in human chronic HBV infection remain incompletely defined.

#### 5.3.3 Immune-mediated non-cytolytic cccDNA degradation

Beyond hepatocyte-intrinsic metabolic and epigenetic remodeling, innate and adaptive immune cytokines secreted by intrahepatic immune cells trigger non-cytolytic cccDNA degradation and transcriptional silencing, representing a critical endogenous mechanism to restrict the persistent viral reservoir without requiring hepatocyte lysis. Importantly, these immune pathways do not operate independently of metabolic regulation but intersect with the metabolic-epigenetic network at multiple levels to shape cccDNA homeostasis.

As a critical innate immune subset liver-enriched, CD56*^bright^* natural killer (NK) cells serve as an early effector arm of non-cytolytic cccDNA regulation. Clinical evidence from chronic HBV patients who switched from entecavir to Peg-IFN-α therapy demonstrates that functional restoration of peripheral CD56*^bright^* NK cells correlates with HBsAg decline (Shi et al., 2018). Furthermore, *in vitro* co-culture models reveal that these activated NK cells contribute to cccDNA reduction, an effect mediated by tumor necrosis factor-α (TNF-α) and interferon-γ (IFN-γ), rather than relying solely on tumor necrosis factor-related apoptosis-inducing ligand (TRAIL)-mediated cytolytic killing (Shi et al., 2018).

The CD8^+^ T cell-derived IFN-γ and TNF-α axis constitutes the core effector arm of adaptive immune-mediated cccDNA control, as evidenced in clinical samples and cell culture models (Xia et al., 2016). Upon cytokine stimulation, hepatocytes upregulate cytidine deaminases, with apolipoprotein B mRNA editing enzyme, catalytic polypeptide-like 3A (APOBEC3A) preferentially induced by interferons and apolipoprotein B mRNA editing enzyme, catalytic polypeptide-like 3B (APOBEC3B) predominantly triggered by lymphotoxin-β receptor (LTβR) activation via nuclear factor kappa B (NF-κB) signaling and miR-138-5p modulation, as demonstrated in cell culture (Bockmann et al., 2019; Faure-Dupuy et al., 2021). These enzymes translocate into the nucleus and are recruited to cccDNA via interaction with the HBc, introducing extensive C-to-U hypermutations across the viral genome a process, a process validated in cell culture, humanized mice, and clinical samples (Lucifora et al., 2014). The mutated genomes are subsequently degraded by interferon-stimulated exonuclease interferon-stimulated gene 20 (ISG20), completing the clearance process, as supported by clinical samples and *in vitro* biochemical assays (Stadler et al., 2021). Notably, *in vitro* data show that this regulatory effect is independent of CD8^+^ T cell cytotoxic killing, and has been proposed to account for partial cccDNA suppression in surviving hepatocytes during the early immune control phase, with potential relevance to chronic HBV infection characterized by T-cell exhaustion (Xia et al., 2016).

In addition to directly inducing cccDNA degradation, immune cytokines modulate hepatocyte metabolic state and epigenetic modifier expression, thereby establishing crosstalk between immune surveillance and the metabolic-epigenetic network. For example, interferon-α (IFN-α) exerts multiple auxiliary restrictive effects at distinct levels. At the transcriptional level, IFN-α reduces the binding of signal transducer and activator of transcription 1 (STAT1) and signal transducer and activator of transcription 2 (STAT2) transcription factors to transcriptionally active cccDNA in cell culture and humanized mouse models (Belloni et al., 2012). Epigenetically, IFN-α modulates KAT2A-mediated succinylation of histone H3K79 on the HBV cccDNA minichromosome, thereby contributing to cccDNA transcriptional silencing and subsequent decay, as shown *in vitro* and in humanized mice (Yuan et al., 2020). Consistently, this epigenetic regulatory pattern is also observed in the duck hepatitis B virus (DHBV) cell culture model, where IFN-α suppresses DHBV cccDNA transcription by reducing H3K9 and H3K27 acetylation in a manner dependent on HDAC activity and *de novo* protein synthesis (Liu et al., 2013). Additionally, IFN-α blocks *de novo* cccDNA biogenesis by inducing the restriction factor MX dynamin-like GTPase 2 (MX2), which downregulates viral RNA synthesis and, indirectly impairs the conversion of rcDNA to cccDNA in primary human hepatocytes and cell culture (Wang J. et al., 2020). IFN-α further amplifies the core degradation pathway by downregulating ubiquitin-conjugating enzyme E2 L3 (UBE2L3) expression to prevent APOBEC3A degradation, consequently promoting cccDNA decay, an effect supported by clinical samples, cell culture models, and primary human hepatocytes (Zhou et al., 2019).

Other inflammatory cytokines also participate in cccDNA regulation. Interleukin-6 (IL-6) silences HBV cccDNA by reducing histone acetylation, decreasing the recruitment of hepatocyte nuclear factor 1α (HNF1α) and HNF4α, and redistributing signal transducer and activator of transcription 3 (STAT3) from cccDNA to host target genes, as demonstrated in cell culture models (Palumbo et al., 2015). Distinct from IFN-driven APOBEC3A/APOBEC3B-mediated cccDNA restriction, transforming growth factor-β (TGF-β) induces the expression of activation-induced cytidine deaminase (AID) expression in hepatocytes, which is recruited to cccDNA through interaction with HBc to introduce extensive C-to-U hypermutations; subsequent uracil-DNA glycosylase (UNG)-dependent uracil excision and apurinic/apyrimidinic (AP) endonuclease activation ultimately trigger irreversible cccDNA fragmentation and degradation *in vitro* (Qiao et al., 2016).

Accumulating evidence indicates that HBV has evolved multiple evasion strategies to counteract immune-driven cccDNA degradation, with some mechanisms targeting both immune and cell-autonomous restriction pathways. Beyond cytokine-induced APOBEC expression, hepatocyte-intrinsic heterogeneous nuclear ribonucleoprotein A2B1 (hnRNPA2B1) also recruits APOBEC3B to cccDNA via recognition of G-quadruplex structures, forming a hepatocyte-intrinsic surveillance axis independent of immune stimulation, as evidenced by cell culture and clinical sample data (Fu et al., 2026). HBx mitigates both immune and cell-autonomous restriction by triggering hnRNPA2B1 polyubiquitination and subsequent proteasomal degradation, thereby sustaining cccDNA persistence as evidenced in cell culture (Fu et al., 2026). In parallel, HBx upregulates heat shock protein family B member 1 (HSPB1), which stabilizes cccDNA by obstructing APOBEC3A/APOBEC3B recruitment while concurrently impairing CD8^+^ T-cell activity, thereby linking innate and adaptive immune evasion as shown in cell culture and humanized mouse models (Yuan et al., 2026). Furthermore, peptide-based disruption of the HBx-HSPB1 interaction effectively promotes cccDNA decay and reinvigorates intrahepatic T-cell responses, revealing a potential therapeutic strategy validated across cell culture, humanized mice, and immunocompetent mice (Yuan et al., 2026).

Beyond direct immune-mediated degradation, the regulation of the cccDNA reservoir likely differs across the clinical phases of chronic HBV infection, immune-tolerant, immune-clearance, inactive-carrier, and HBeAg-negative infection, shaped by HBeAg serostatus, host immune pressure, hepatic inflammation and fibrosis, metabolic dysfunction such as hepatic steatosis, and antiviral treatment (Cheng et al., 2011; Lebossé et al., 2020; Magri et al., 2022; Suslov et al., 2021; Testoni et al., 2024; Werle-Lapostolle et al., 2004; Xia et al., 2016). Indeed, in nucleos(t)ide-naïve patients with chronic hepatitis B, baseline intrahepatic cccDNA levels correlate with necroinflammation and fibrosis, and treatment-induced reductions in cccDNA are linked to histological improvement (Cheng et al., 2011). Whether this phase-divergent cccDNA behavior is driven by metabolic-epigenetic regulatory pathways remains unclear. In the HBeAg-positive high-replication phase, both cccDNA transcription and peak, coinciding with the highest serum HBV DNA levels (Thi et al., 2017). Epigenetic silencing of cccDNA may underlie the HBeAg-negative infection phase with substantially improved immune control and strong transcriptional suppression of the cccDNA reservoir (Vanwolleghem et al., 2022). In inactive carriers (HBeAg-negative chronic HBV infection), silencing of cccDNA transcriptional activity leads to a low-replication carrier state with an excellent long-term prognosis (Tu et al., 2022a). Notably, hepatic steatosis is clinically associated with reduced HBV replication (Huang et al., 2023). Overall, most phase-specific observations are derived from serum biomarker correlations rather than direct intrahepatic metabolic-epigenetic profiling. Causal evidence that metabolic-epigenetic pathways drive phase-specific cccDNA behavior in humans is still lacking, representing a key gap for future clinical studies.

Collectively, these observations position the metabolic-epigenetic axis as a central, integrated regulator of cccDNA reservoir homeostasis rather than a collection of independent molecular interactions. Changes in hepatic physiology remodel intracellular metabolite availability, which in turn modulates histone PTMs and reconfigures the chromatin state of the cccDNA minichromosome, governing its transcriptional activity and long-term stability. Within this cascade, liver-enriched nuclear receptors act as upstream sensors coupling metabolic fluctuations to epigenetic reprogramming, while immune cytokines provide an additional regulatory layer via non-cytolytic cccDNA silencing and degradation, together adapting the viral reservoir to the hepatic microenvironment. Nevertheless, most observations derive from reductionist cellular systems or non-physiological infected animal models, and their quantitative relevance to chronic human HBV infection remains incompletely validated. Many pathways also exert context-dependent effects that vary with disease stage, coexisting liver pathology, and antiviral treatment. Moreover, because most studies have examined individual metabolites or epigenetic marks in isolation, the integrated behavior of the full metabolic-epigenetic network in the intact human liver remains poorly characterized. Resolving these knowledge gaps is critical for designing curative therapies that target metabolic dependencies of the HBV reservoir (Figure 3).

**FIGURE 3:**
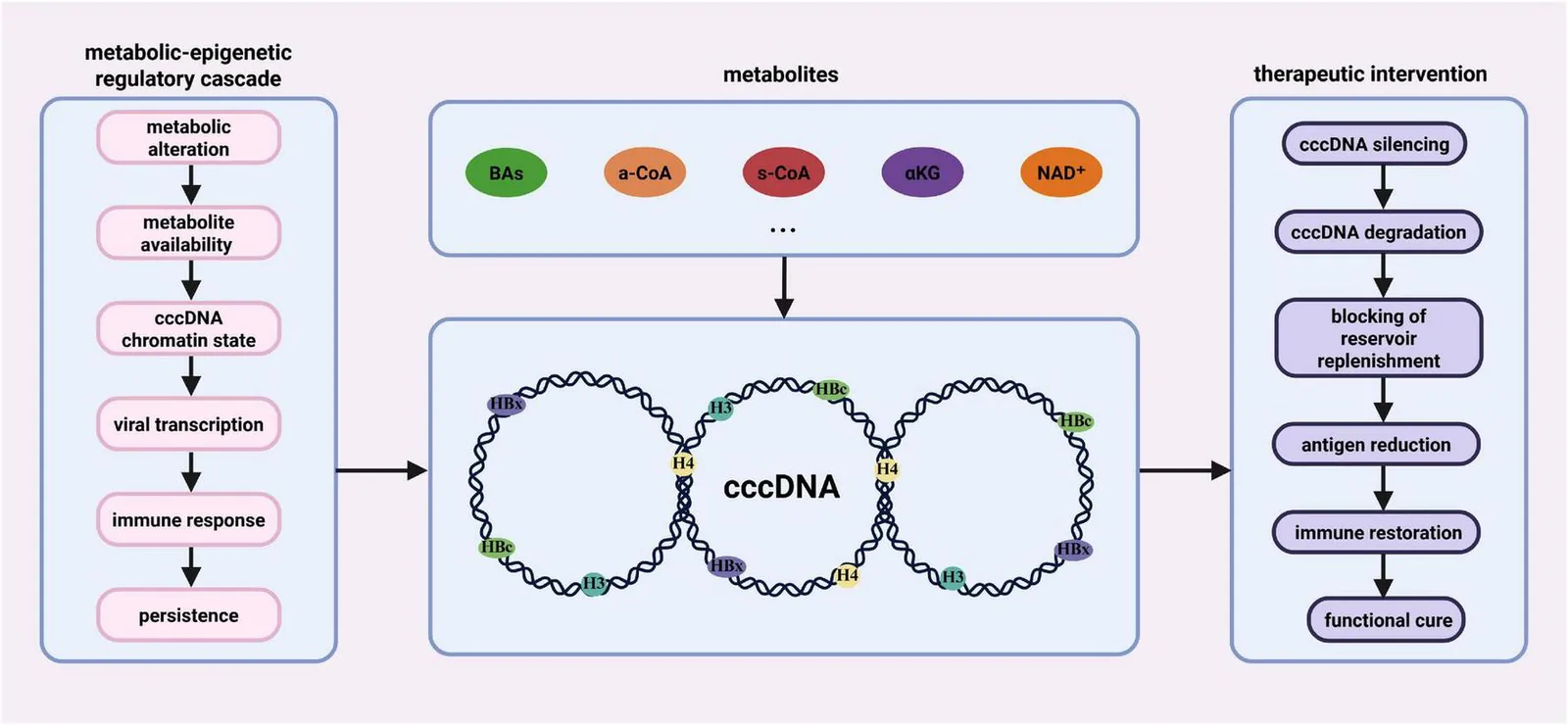
Conceptual schematic illustrating the metabolic-epigenetic regulation of the HBV cccDNA reservoir and the corresponding sequential therapeutic strategies. Host metabolic alterations shape the availability of metabolites, including BAs, α-CoA, s-CoA, αKG, and NAD^+^, which modulate the chromatin state of the nuclear cccDNA minichromosome and thereby govern viral transcription, antiviral immune responses and eventual viral persistence. Therapeutic interventions are designed to target distinct nodes of this cascade in a stepwise manner, proceeding from cccDNA silencing and cccDNA degradation to blocking reservoir replenishment, followed by antigen reduction and immune restoration, toward the ultimate goal of functional cure. BAs, bile acids; α-CoA, acetyl coenzyme A; s-CoA, succinyl coenzyme A; αKG, α-ketoglutarate; NAD^+^: nicotinamide adenine dinucleotide. (This Figure was created in https://BioRender.com).

### 5.4 cccDNA pool homeostasis and intracellular amplification

#### 5.4.1 Mature nucleocapsid re-import and intracellular cccDNA amplification

Following reverse transcription within the cytoplasmic nucleocapsids, mature capsids containing rcDNA are directed toward divergent fates, either being enveloped and secreted as progeny virions, or routed back to the nucleus to amplify the intracellular cccDNA pool (Wei and Ploss, 2021b). This intracellular amplification pathway is a hallmark of hepadnaviruses and is fundamental to the establishment and maintenance of chronic HBV infection (Block et al., 2007). Guided by the importin-α(IMPα)/importin-β (IMPβ) complex, the nucleocapsid is transported along microtubules to the NPC, where it interacts with Nup153 in the nuclear basket and undergoes disassembly to release rcDNA into the nucleoplasm (Chuang et al., 2022; Guo and Guo, 2015; Guo et al., 2017). After nuclear delivery, rcDNA is converted back into cccDNA, recapitulating the initial step of infection. In naturally infected hepatocytes, cccDNA copy numbers are highly heterogeneous, typically ranging from a few to tens of copies per cell and occasionally exceeding 50 copies, with values approaching 200 copies per cell reported in patients with chronic hepatitis B (Guo and Guo, 2015). This self-renewing loop ensures that, even in the absence of continuous *de novo* infection, infected hepatocytes retain a stable, self-sustaining cccDNA reservoir.

#### 5.4.2 Homeostatic regulation of the cccDNA pool

To prevent excessive intracellular accumulation that could trigger host innate immune sensing and hepatocyte injury, the cccDNA pool is subject to tight homeostatic regulation maintained by a dynamic equilibrium among intracellular amplification, molecular decay, and epigenetic transcriptional silencing. First, the routing of mature cytoplasmic nucleocapsids toward either virion secretion or nuclear translocation is dictated by nucleocapsid maturation status and host cellular factors, thereby limiting excessive rcDNA nuclear import and unregulated cccDNA amplification. Second, although cccDNA exhibits high intrinsic stability with a half-life ranging from 33 to 50 days in woodchuck hepatitis B virus (WHV) models and 35–57 days in DHBV models (Addison et al., 2002; Zhu et al., 2001), the episomal viral genome is not permanently retained in hepatocytes. Notably, these cccDNA half-life parameters are derived from woodchuck and duck hepadnavirus models; precise turnover kinetics in human chronically infected livers remain technically challenging to quantify. As elaborated in preceding sections, multiple non-cytolytic degradation pathways dominated by the APOBEC3 enzyme family actively drive cccDNA clearance; meanwhile, IFN-α and other intrahepatic immune cytokines, together with viral and metabolic regulatory cues, mediate transcriptional silencing of the cccDNA minichromosome via epigenetic chromatin remodeling. Third, owing to its episomal nature and absence of centromeric sequences required for mitotic segregation, cccDNA undergoes passive dilution and gradual loss during hepatocyte proliferation (Dandri and Petersen, 2016; Lin et al., 2025; Yan et al., 2019). This multi-layered balance between *de novo* intracellular cccDNA amplification and coordinated clearance, silencing and passive dilution sustains the lifelong persistence of the intrahepatic cccDNA reservoir, which constitutes the primary obstacle to achieving a sterilizing cure of chronic HBV infection.

## 6 Virion maturation, secretion and viral dissemination

Following nuclear export, cytoplasmic pgRNA supports viral protein translation and reverse transcription to generate mature nucleocapsids containing rcDNA (Ding et al., 2021). During HBV replication, these mature capsids follow two distinct routes. Most capsids are enveloped and secreted as infectious Dane particles or non-infectious SVPs to facilitate viral dissemination (Blanchet and Sureau, 2006; Komla-Soukha and Sureau, 2006; Wang et al., 2024). A subset of mature rcDNA-containing capsids re-enters the nucleus to replenish the cccDNA reservoir (Diogo Dias et al., 2021; Figure 1).

This intracellular recycling mechanism sustains cccDNA homeostasis and viral persistence, independently of *de novo* exogenous infection, constituting a central mechanism for maintaining the persistent HBV reservoir (Cui et al., 2015). Notably, virion secretion and SVP overproduction primarily promote immune evasion and persistent antigenemia, while cell-to-cell transmission expands the pool of infected hepatocytes, neither process directly impinging on the metabolic or epigenetic regulation of cccDNA (Goyal and Murray, 2016; Liu et al., 2025; Rexhouse et al., 2025; Salimzadeh et al., 2018). Nevertheless, the quantitative contribution of cell-to-cell transmission to intrahepatic reservoir maintenance in HBV-infected patients remains incompletely resolved, as most functional data are obtained from *in vitro* co-culture systems. Importantly, while virion secretion and cell-to-cell spread shape the size and distribution of the infected hepatocyte population, the transcriptional activity and long-term stability of the cccDNA reservoir itself are ultimately governed by the host metabolic-epigenetic microenvironment.

## 7 Therapeutic strategies

While current NAs effectively suppress HBV replication, they fail to eliminate the cccDNA reservoir or block the secretion of immunosuppressive SVPs. Achieving a functional cure necessitates combinatorial regimens that address the four core biological barriers to viral persistence. The first barrier is transcriptionally active and stable cccDNA minichromosomes. The second is continuous *de novo* cccDNA formation and intrahepatic viral spread. The third is persistent antigenemia driving immune tolerance. The fourth is integrated HBV DNA as an independent source of HBsAg. The following therapeutic strategies are organized according to the core biological barriers that each intervention targets to overcome HBV persistence (Figure 3 and Table 1).

**TABLE 1:** Therapeutic strategies for HBV cure categorized by core biological barriers to viral persistence.

Agent	Category	Class	Compounds	Mechanism	Stage	References
Silencing and eliminating intrahepatic cccDNA reservoir	Transcriptional silencing	FXR agonists	GW4064	Repressing cccDNA transcription and reducing pgRNA synthesis	Preclinical	Mouzannar et al., 2019
			Vonafexor	Repressing cccDNA transcription and pgRNA synthesis	Phase Ib	Erken et al., 2021
		RXR agonist	Bexarotene	Suppressing HBV infection across multiple models via RXRα activation, coupling HBV restriction to hepatic lipid metabolism and the AA/eicosanoid pathway	Preclinical	Song et al., 2018
		Epigenetic modulator	C646	p300/CBP inhibitor; diminishing cccDNA-associated H3K27 acetylation	Preclinical	Tropberger et al., 2015
			Tune-401	Durably repressing cccDNA and integrated DNA transcription	Phase Ib	Chang et al., 2026
		Metabolic regulator	Bempedoic acid	ATP citrate lyase inhibitor; reducing acetyl-CoA pools and global H3K27 acetylation	Preclinical	Li et al., 2026; Ferri et al., 2024; Sivanand et al., 2017
			Glycine	Stimulating hepatocyte proliferation via GlyT1/mTORC1 to passively dilute cccDNA	Preclinical	Lin et al., 2025
	Direct cccDNA cleavage	Meganuclease	ARCUS-POL	ARCUS nuclease; DSB-mediated cccDNA linearization and mutagenic inactivation	Preclinical	Gorsuch et al., 2022
			PBGENE-HBV	ARCUS nuclease; site-specific mutagenesis to inactivate episomal cccDNA	Phase I	Kozlov et al., 2025
		Cytosine base editor	BE4	Introducing premature stop codons within cccDNA without double-strand breaks	Preclinical	Smekalova et al., 2024
		CRISPR-Cas9	Cas9/gRNA	VSV-G engineered extracellular vesicles deliver Cas9 to cleave cccDNA/integrated DN A	Preclinical	Zeng et al., 2024
	Immune-mediated degradation	–	IFN-α	Upregulating APOBEC3A/ISG20; inducing C-to-U hypermutation and degradation	Approved	Lucifora et al., 2014
			LTβR agonists	Upregulating APOBEC3B; inducing cccDNA deamination and degradation	Preclinical	Lucifora et al., 2014
			UNG inhibitors	Inhibiting UNG to preserve uracil lesions and enhancing APOBEC-driven cccDNA hypermutation	Preclinical	Kitamura et al., 2022
Blocking *de novo* cccDNA formation and intrahepatic viral spread	Entry inhibitor	NTCP inhibitor	Bulevirtide	NTCP receptor inhibitor; blocking cell-free virion entry	Preclinical	Ni et al., 2014
	Intracellular amplification inhibitors	CAM, Class II	JNJ-56136379	Binds HBc; disrupting early/late life cycle, preventing pgRNA encapsidation	Phase IIb	Berke et al., 2020; Yuen et al., 2023
		CAM, Class I	ABI-H0731	Binds dimer-dimer interface; accelerating aberrant assembly, blocking uncoating	Phase II	Huang et al., 2020
		CAM-E	GST-HG141	Stabilizing capsid structure; hindering uncoating	Phase II	Wang et al., 2025
			ALG-000184	ALG-001075 prodrug; blocking capsid assembly, pgRNA encapsidation and cccDNA establishment	Phase II	Yuen et al., 2026
Suppressing antigenemia and restoring immune control	HBsAg secretion inhibition	NAPs	REP 2139-Mg	Blocking SVP release from hepatocytes	Phase II	Bazinet et al., 2020
		REP 2165-Mg	Blocking SVP release from hepatocytes	Phase II	Bazinet et al., 2020
		Small-molecule inhibitor	GST-HG131	Blocks HBsAg release from hepatocytes	Phase Ia	Li et al., 2022
	Transcript-targeted silencing	ASO	Bepirovirsen	Degrading HBV transcripts derived from cccDNA and integrated HBV DNA	Phase III	Hou et al., 2026
		GalNAc-siRNA+ antibody	VIR-2218+VIR-3434	Knocking down viral transcripts to reduce antigen production and blocking reinfection	Phase II	Jeng and Lok, 2023
		GalNAc-siRNA	JNJ-73763989	Targeting multiple HBV RNA species to reduce viral protein production including HBsAg	Phase IIb	Yuen et al., 2023
	Immune restoration	TLR7 agonist	Vesatolimod	TLR7 agonist; activating innate immunity	Phase II	Agarwal et al., 2018
		Therapeutic vaccine	VTP-300	Inducing HBV-specific CD4^+^ and CD8^+^ T-cell responses	Phase Ib/IIa	Tak et al., 2024
		Anti-PD-1 mAb	Nivolumab	Blocks PD-1/PD-L1 axis, reinvigorating exhausted HBV-specific T cells	Phase Ib	Gane et al., 2019
		TCR-T adoptive therapy	SCG101	Autologous TCR-T cells targeting HBV peptides to clear infected hepatocytes	Phase I/II	Wu et al., 2025

HBV, hepatitis B virus; cccDNA, covalently closed circular DNA; FXR, farnesoid X receptor; pgRNA, pregenomic RNA; RXR, retinoid X receptor; RXRα, retinoid X receptor alpha; AA, arachidonic acid; p300/CBP, p300/CREB-binding protein; H3K27, histone H3 lysine 27; ATP, adenosine triphosphate; acetyl-CoA, acetyl coenzyme A; GlyT1, glycine transporter 1; mTORC1, mammalian target of rapamycin complex 1; DSB, double-strand break; CRISPR-Cas9, clustered regularly interspaced short palindromic repeats and CRISPR-associated protein 9; gRNA, guide RNA; VSV-G, vesicular stomatitis virus glycoprotein G; IFN-α, interferon alfa; APOBEC3A, apolipoprotein B mRNA-editing catalytic polypeptide-like 3A; ISG20, interferon-stimulated gene 20; LTβR, lymphotoxin beta receptor; APOBEC3B, apolipoprotein B mRNA-editing catalytic polypeptide-like 3B; UNG, uracil-DNA glycosylase; NTCP, sodium taurocholate cotransporting polypeptide; CAM, capsid assembly modulator; HBc, hepatitis B core protein; CAM-E, capsid assembly modulator-empty capsid; NAPs, nucleic acid polymers; SVP, subviral particle; HBsAg, hepatitis B surface antigen; ASO, antisense oligonucleotide; GalNAc-siRNA, N-acetylgalactosamine-conjugated small interfering RNA; PD-1, programmed cell death protein 1; PD-L1, programmed cell death ligand 1; TCR-T, T-cell receptor-engineered T cell.

### 7.1 Silencing and eliminating the intrahepatic cccDNA reservoir

The cccDNA minichromosome is the central reservoir of HBV persistence. Therapeutic strategies in this category aim either to transcriptionally silence cccDNA via metabolic and epigenetic reprogramming, or to directly degrade or mutate cccDNA to achieve permanent inactivation.

#### 7.1.1 Transcriptional silencing via metabolic-epigenetic reprogramming

The dependence of cccDNA on host metabolic intermediates and epigenetic modifiers reveals actionable therapeutic nodes for silencing the viral minichromosome without direct viral targeting.

##### 7.1.1.1 Nuclear receptor modulators

Liver-enriched nuclear receptors such as FXR, PPARα, and HNF4α govern both hepatic metabolism and cccDNA transcription. For instance, FXR agonists such as GW4064 repress cccDNA transcription and reduce pgRNA synthesis, effectively silencing the viral minichromosome (Mouzannar et al., 2019). A phase Ib trial of the non-BA selective FXR agonist vonafexor (EYP001) demonstrated that while 4-week monotherapy yielded only a modest HBsAg decline without reducing HBV DNA, its combination with Peg-IFN-α2a synergistically suppressed cccDNA transcriptional markers including hepatitis B core-related antigen (HBcrAg) and pgRNA, confirming that pharmacological FXR modulation can transcriptionally silence the viral reservoir (Erken et al., 2021). Unlike the inhibitory effect triggered by FXR, PPARα heterodimerizes with RXRα to bind regulatory elements within the HBV genome and augment viral transcription during established infection. Interestingly, RXRα itself acts as a host restriction factor during *de novo* infection, with its depletion promoting cccDNA formation, whereas the RXR agonist bexarotene suppresses HBV infection across multiple models (Song et al., 2018). Mechanistically, RXRα couples HBV restriction to hepatic lipid metabolism such that its silencing downregulates the arachidonic acid (AA)/eicosanoid biosynthetic pathway, while exogenous AA treatment phenocopies RXRα activation in inhibiting infection (Song et al., 2018). Nuclear receptor modulators are in early clinical development, and FXR agonists represent a class of agents in this category. A key limitation is that these agents primarily reduce cccDNA transcription rather than eliminating the reservoir. Monotherapy typically achieves only modest and transient HBsAg reduction, and long-term off-treatment durability remains unproven. Additionally, nuclear receptors have broad physiological roles in hepatic metabolism. These broad physiological roles raise concerns regarding systemic metabolic side effects during chronic administration.

##### 7.1.1.2 Epigenetic intervention

cccDNA chromatin compaction is dynamically regulated by histone PTMs. Histone acetylation generally promotes transcription, whereas methylation exerts site-specific effects, with H3K4 or H3K36 methylation associated with transcriptional activation, while H3K9 and H3K27 methylation are hallmarks of gene silencing and chromatin condensation (Pan et al., 2015). Therapeutic strategies aimed at cccDNA silencing focus on enhancing repressive marks or reducing activating modifications. Histone acetyltransferase inhibitors, such as the p300/CBP inhibitor C646, diminish cccDNA-associated H3K27 acetylation and suppress cccDNA transcription in primary human hepatocytes (Tropberger et al., 2015). Histone deacetylase inhibitor treatment can trigger HBV reactivation in latently infected patients (Ritchie et al., 2009), consistent with the model that elevated histone acetylation at the cccDNA minichromosome drives viral transcription (Pollicino et al., 2006). This risk of non-selective epigenetic agents in clinical settings does not negate the therapeutic potential of cccDNA-targeted epigenetic-modifying strategies.

Beyond exogenous pharmacological agents, endogenous host factors and non-coding RNAs dynamically reshape the cccDNA epigenome. For instance, Lamin A/C stabilizes HBx to maintain active marks (H3K4 trimethylation and H3K27 acetylation), and its knockdown triggers HBx degradation and shifts the minichromosome toward repressive marks (of H3K9 trimethylation and H3K27 trimethylation) (Jiang et al., 2025). Conversely, the long non-coding RNA (lncRNA) HOXA cluster antisense RNA 2 (HOXA-AS2) recruits the metastasis-associated protein 1 (MTA1)-HDAC1/2 deacetylase complex to cccDNA, directly erasing H3K9 acetylation and H3K27 acetylation to transcriptionally silence the viral reservoir (Qin et al., 2024). Beyond the classical acetylation/methylation binary, metabolism-linked histone acylations have emerged as a new regulatory layer. Given that KAT2A catalyzes H3K79 succinylation to enhance cccDNA transcription (Qin et al., 2021), pharmacological inhibition of its succinyltransferase activity represents a promising strategy to reverse this permissive epigenetic state and silence the viral reservoir. Conversely, therapeutic strategies aimed at restoring or leveraging the NAD^+^-dependent desuccinylase activity of SIRT7 could reinforce cccDNA silencing by removing the activating H3K122 succinylation mark (Yu et al., 2021). Beyond succinylation, H4K8 2-hydroxyisobutyrylation is confirmed to modulate cccDNA activity (Zhao et al., 2022), indicating that intermediary metabolites serve as direct epigenetic effectors. At the chromatin-organizational level, the SMC5/6 complex restricts cccDNA transcription by compacting the viral minichromosome, but HBx antagonizes this restriction by targeting SMC5/6 for degradation, while IFNα partially restores cellular SMC5/6 abundance and this effect is further potentiated by HBx-targeting small interfering RNA (siRNA), with the combination suppressing viral markers by over 95% (Hu et al., 2024). Tune-401, an epigenetic silencer designed to durably repress cccDNA and integrated DNA, has entered phase Ib clinical evaluation for targeting cccDNA-driven viral activity (Chang et al., 2026). Most epigenetic strategies remain at the preclinical stage. First, there is a lack of cccDNA-specific targeting, and broad epigenetic modifiers carry risks of off-target host gene regulation and hepatotoxicity. Second, silencing is typically reversible and requires sustained drug exposure. Third, epigenetic monotherapy cannot eliminate cccDNA and is unlikely to achieve durable HBsAg loss alone.

##### 7.1.1.3 Disrupting the metabolic-epigenetic cccDNA niche

Given the critical dependence of cccDNA on host metabolic intermediates and epigenetic modifiers, disrupting this maintenance network offers a promising strategy to drive the viral reservoir toward functional exhaustion and passive dilution. Inhibiting acetyl-CoA generation represents a primary strategy. Pharmacological agents such as the ATP citrate lyase inhibitor bempedoic acid, or interventions disrupting the acetyl-CoA-generating enzyme acyl-CoA synthetase short-chain family member 2 (ACSS2), can markedly diminish cellular acetyl-CoA pools and blunt global H3K27 acetylation modification, thereby theoretically suppressing cccDNA transcription (Ferri et al., 2024; Li et al., 2026; Sivanand et al., 2017). Furthermore, manipulating central carbon metabolism to alter the availability of epigenetic cofactors offers a novel intervention. Nutrient-derived modulators, such as glycine, stimulate hepatocyte proliferation via glycine transporter-1 (GlyT1) and mammalian target of rapamycin complex 1 (mTORC1) activation, accelerating the passive dilution of cccDNA, and its combination with IFN-α significantly enhances antiviral efficacy (Lin et al., 2025). Similarly, inhibiting the succinyltransferase activity of KAT2A holds potential to reverse the transcriptionally permissive epigenetic landscape of cccDNA, pushing the viral reservoir toward silencing and eventual decay (Qin et al., 2021). Metabolic interventions are largely preclinical and mechanistic. Their clinical translation faces two key challenges. First, metabolic perturbation is non-specific and may alter global hepatic physiology. Second, their effect on cccDNA is indirect and relatively weak, making them more suitable as adjunctive rather than curative monotherapies. Notably, these metabolic-epigenetic-targeting approaches predominantly modulate the transcriptional state of cccDNA within already-infected hepatocytes and do not prevent *de novo* infection. Consequently, combinatorial regimens incorporating entry- or transmission-blocking agents are required to constrain continuous new cccDNA formation.

#### 7.1.2 Direct cccDNA cleavage and mutagenesis

Physical elimination or permanent mutagenic inactivation of cccDNA represents the ultimate goal of a sterilizing cure. Clustered regularly interspaced short palindromic repeats (CRISPR) and CRISPR-associated (Cas) systems, base editors, and engineered nucleases enable direct targeting and mutational disruption of HBV cccDNA.

When delivered systemically as lipid nanoparticle (LNP)-encapsulated mRNA, the sequence-specific ARCUS meganuclease (ARCUS-POL) reduces cccDNA levels in infected primary human hepatocytes and mediates efficient on-target editing in mouse and non-human primate models, durably decreasing circulating HBsAg in mice without detectable chromosomal translocations (Gorsuch et al., 2022). Building on these preclinical data, PBGENE-HBV has recently advanced into the first-in-human gene editing trial for chronic HBV infection, with early clinical data demonstrating notable HBsAg reduction following a single systemic dose (Kozlov et al., 2025). Cytosine base editors including BE4 cytosine base editor, which introduce premature stop codons within cccDNA without triggering DNA double-strand breaks, achieve robust modification of episomal cccDNA and integrated HBV DNA in infected primary human hepatocytes upon LNP delivery, yielding sustained suppression of viral markers in mouse models (Smekalova et al., 2024). Beyond LNP delivery, engineered extracellular vesicles incorporating vesicular stomatitis virus G protein (VSV-G) have been developed to package functional Cas9/guide RNA (gRNA), enabling endosomal escape and leading to pronounced mutational inactivation of episomal cccDNA and integrated HBV DNA fragments in HBV-replicating and transgenic mouse models (Zeng et al., 2024).

Gene editing represents a direct strategy with the potential to permanently inactivate cccDNA, but it remains at an early translational stage. Only one meganuclease has advanced to first-in-human trials. Major unresolved challenges include several aspects. First, efficient delivery of editing machinery to quiescent hepatocytes *in vivo* remains difficult. Second, there is risk of off-target editing of the host genome. Third, these strategies cannot eliminate integrated HBV DNA fragments, which may continue to produce HBsAg and hinder functional cure endpoints. Fourth, there is potential immune recognition of edited hepatocytes or bacterial-derived nucleases.

#### 7.1.3 Immune-mediated non-cytolytic cccDNA degradation

Innate and adaptive immune pathways drive non-cytolytic cccDNA clearance. Stimulation with IFN-α or LTβR agonists upregulates APOBEC3A and APOBEC3B, respectively, which directly deaminate cccDNA through interaction with the HBc, selectively driving extensive C-to-U hypermutation and degradation of the viral genome while sparing host DNA (Lucifora et al., 2014). Subsequent work further clarified that APOBEC3A-mediated deamination requires cooperating nucleases exemplified by ISG20 to achieve efficient cccDNA degradation (Stadler et al., 2021). Interestingly, while UNG counteracts lethal viral mutagenesis by removing uracil bases generated by cytidine deamination, thereby partially safeguarding cccDNA integrity, its pharmacological inhibition has been shown to enhance IFN-γ-mediated hypermutation and suppress viral rebound, highlighting the complex interplay between host editing enzymes and repair pathways in determining cccDNA fate (Kitamura et al., 2022). IFN-α is the only clinically approved agent in this category, but its efficacy is modest and highly variable among patients, and it is associated with significant systemic side effects. LTβR agonists and UNG inhibitors remain in preclinical development. A fundamental limitation is that immune-mediated degradation requires robust intrahepatic immune function, which is often impaired in chronic HBV infection. Additionally, the pathway does not efficiently clear all cccDNA molecules, and residual transcriptionally competent cccDNA can drive viral rebound after treatment cessation. Questions regarding how this machinery discriminates viral episomal DNA from host chromatin also remain incompletely resolved.

### 7.2 Blocking *de novo* cccDNA formation and intrahepatic spread

Even if existing cccDNA is silenced or cleared, continuous *de novo* cccDNA formation via cell-free virion entry, cell-to-cell transmission, and intracellular nucleocapsid re-import can replenish the reservoir. Strategies in this category aim to block new cccDNA biogenesis.

#### 7.2.1 Viral entry inhibition

The NTCP inhibitor bulevirtide blocks HBV adsorption to hepatocytes and reduces *de novo* infection (Ni et al., 2014). The agent has received regulatory approval in the European Union for chronic hepatitis delta virus (HDV) infection (Sauter et al., 2021) and is being evaluated in HBV. Its key limitation is that it only blocks cell-free virion entry and has no effect on cccDNA already established in hepatocytes or on NTCP-independent cell-to-cell transmission.

#### 7.2.2 Blocking cell-to-cell transmission

Cell-to-cell transmission enables HBV to evade extracellular immune surveillance and spread across neighboring hepatocytes. While bulevirtide blocks cell-free virion entry by competing for NTCP receptor binding, it cannot intercept NTCP-independent intercellular viral propagation, as evidenced by sustained cell-to-cell spread under high-dose bulevirtide treatment in HDV infection models, and exerts no influence on viral production and release within already-infected hepatocytes (Zhang et al., 2022). Host restriction factors, such as the interferon-stimulated genes ISG20 and tetherin, naturally constrain HBV dissemination via viral RNA degradation and suppression of virion release (Leong et al., 2016; Yan et al., 2015). There are currently no clinically approved agents that specifically block HBV cell-to-cell transmission. Most mechanistic data come from *in vitro* co-culture systems, and the quantitative contribution of cell-to-cell spread to intrahepatic reservoir maintenance in patients remains incompletely defined.

#### 7.2.3 Inhibiting intracellular cccDNA amplification

Capsid assembly modulators (CAMs) disrupt nucleocapsid maturation, preventing virion release and limiting intracellular amplification of the cccDNA pool (Bassit et al., 2023). The CAM Class II JNJ-56136379 primarily binds to the HBc and disrupts both early and late stages of the HBV life cycle by modulating capsid assembly dynamics, preventing pgRNA encapsidation and suppressing HBV DNA replication (Berke et al., 2020). However, phase IIb results showed limited efficacy for JNJ-56136379 plus NAs, failing to meet stopping criteria or show synergy with siRNA (Yuen et al., 2023). Similarly, ABI-H0731 is a direct-acting HBc inhibitor that binds to the capsid protein dimer-dimer interface and accelerates aberrant capsid assembly, thereby blocking pgRNA encapsidation and subsequent HBV DNA replication, while also disrupting incoming nucleocapsids to cause premature release of rcDNA before nuclear delivery, ultimately preventing *de novo* cccDNA establishment (Huang et al., 2020). Capsid assembly modulators-empty (CAM-E) agent GST-HG141 binds to HBcAg, stabilizing the viral capsid structure and hindering the uncoating process, thereby reducing newly formed rcDNA and potentially contributing to the decrease in the cccDNA reservoir (Wang et al., 2025). Phase II clinical data further demonstrate that GST-HG141 effectively reduces residual HBV DNA and pgRNA in NAs-treated chronic hepatitis B (CHB) patients with low-level viremia (Wang et al., 2025). In addition, ALG-000184, a CAM-E prodrug, achieved potent HBV DNA declines with good tolerability in phase I and is now in phase II (Yuen et al., 2026). Collectively, these agents misdirect capsid assembly, reducing the pool of mature rcDNA available for nuclear import and subsequent cccDNA replenishment. However, CAM monotherapy typically does not reduce HBsAg or cccDNA levels substantially, because these agents do not eliminate pre-existing cccDNA. Their primary clinical role is to block intracellular cccDNA amplification and reduce viral spread, and they require combination with cccDNA-silencing or immune-based strategies.

### 7.3 Suppressing antigenemia and restoring immune control

Persistent high-level circulating HBsAg and other viral antigens drive T-cell exhaustion and immune tolerance, which is a major barrier to functional cure. Strategies in this category aim to reduce antigen load and reconstitute antiviral immunity.

#### 7.3.1 Inhibition of HBsAg secretion

Inhibiting HBsAg secretion represents a direct-acting antiviral paradigm aimed at clearing circulating antigens. Nucleic acid polymers (NAPs), such as REP 2139 and REP 2165, function by blocking SVP release from hepatocytes (Inoue et al., 2021). REP 2139-Mg and REP 2165-Mg block secretion of HBV SVPs, and their combination with tenofovir disoproxil fumarate (TDF) and Peg-IFN yields marked HBsAg decline in HBeAg-negative chronic HBV patients in a phase II open-label trial (Bazinet et al., 2020). Beyond oligonucleotide-based agents, emerging small molecule HBsAg inhibitors such as GST-HG131 are currently in early clinical development, offering the prospect of oral therapies to disrupt antigenemia (Li et al., 2022). NAPs are clinically HBsAg secretion inhibitors, and phase II data show high rates of HBsAg reduction. However, these agents do not reduce cccDNA or integrated DNA. HBsAg suppression is often transient after treatment cessation, and long-term durability of functional cure remains to be confirmed. Small-molecule HBsAg inhibitors are still in early development.

#### 7.3.2 Transcript-targeted silencing of viral antigens

Antisense oligonucleotides (ASOs) and N-acetylgalactosamine (GalNAc)-conjugated siRNAs provide a finite-duration approach to suppress all viral transcripts derived from both cccDNA and integrated HBV DNA, thereby removing immunosuppressive antigens and exposing infected cells to immune clearance.

In two phase III trials, 24-week bepirovirsen treatment achieved functional cure in 20% and 19% of virally suppressed patients receiving NAs, with responses maintained for 24 weeks after NA discontinuation and an acceptable safety profile, establishing bepirovirsen as a first-in-class ASO capable of targeting the entire HBV transcriptome (Hou et al., 2026). Building on this, RNA interference strategies have advanced into combination regimens with neutralizing antibodies to deepen and accelerate HBsAg decline while blocking reinfection. In a phase II study, treatment with GalNAc-siRNA VIR-2218 and VIR-3434 for 12–20 weeks led to end-of-treatment HBsAg levels below 10 IU/mL in most participants; however, HBsAg seroclearance was not observed in any individual (Jeng and Lok, 2023). Similarly, in the phase IIb trial, the GalNAc-siRNA JNJ-73763989 plus NAs dose-dependently reduced HBsAg but rarely achieved HBsAg seroclearance (Yuen et al., 2023). Antisense oligonucleotides are transcript-targeted agents, and data demonstrate durable functional cure in a subset of patients. Key limitations include several aspects. First, repeated dosing is required for sustained efficacy. Second, there is potential for off-target effects and hepatotoxicity. Third, these agents do not eliminate cccDNA, so viral rebound remains possible if treatment is stopped before immune control is established.

#### 7.3.3 Immune reconstitution and adoptive cellular immunotherapy

Separate phase II trials of vesatolimod assessed monotherapy in virally suppressed chronic HBV patients and combination therapy with tenofovir in treatment-naive viremic patients, and both trials demonstrated dose-dependent induction of interferon-stimulated genes without clinically meaningful HBsAg reduction (Agarwal et al., 2018; Janssen et al., 2018). VTP-300 is a ChAdOx1/modified vaccinia virus Ankara (MVA) prime-boost therapeutic vaccine encoding HBV antigens that elicited potent HBV-specific CD4^+^ and CD8^+^ T-cell immune responses. Phase Ib/IIa data showed that VTP-300 alone or combined with low-dose nivolumab reduces HBsAg in selected patients with low baseline HBsAg, with a favorable safety profile (Tak et al., 2024). In a phase Ib study, nivolumab monotherapy was well tolerated and led to HBsAg declines in most virally suppressed HBeAg-negative patients, with sustained HBsAg loss observed in one patient (Gane et al., 2019). These immune modulatory approaches produce variable responses and often only achieve limited antigen reduction in a subset of treated patients. Distinct from the non-cytolytic editing mechanisms described above, adoptive cellular therapies aim to physically eliminate infected or transformed hepatocytes expressing HBV antigens. SCG101 is an autologous therapy engineered to express a stable, high-affinity T-cell receptor (TCR) that specifically recognizes HBV peptides presented by human leukocyte antigen (HLA)-A*02:01, demonstrating a manageable safety profile and preliminary on-target efficacy in HBV-related hepatocellular carcinoma patients, evidenced by significant T-cell expansion, rapid HBsAg decline, and objective tumor regression (Wu et al., 2025). Adoptive T-cell therapy is in early clinical development, and it is currently focused on HBV-related hepatocellular carcinoma rather than chronic hepatitis B. Major barriers include HLA restriction, manufacturing complexity, and risk of liver injury from immune-mediated hepatocyte destruction. For chronic HBV, the broader challenge remains restoring endogenous immune tolerance without triggering severe liver inflammation.

Collectively, the therapeutic landscape for chronic HBV has expanded dramatically, but no single agent addresses all biological barriers simultaneously. Several cross-cutting challenges remain. First, integrated HBV DNA persists as a major obstacle. Most strategies that silence or degrade episomal cccDNA cannot eliminate integrated HBV DNA fragments. These fragments can serve as an independent source of HBsAg and limit the achievement of functional cure, particularly in HBeAg-negative patients. Second, there is a lack of cccDNA-specific biomarkers. There is no gold-standard non-invasive biomarker to directly quantify intrahepatic cccDNA and distinguish transcriptionally silent from transcriptionally competent cccDNA. This gap hinders real-time evaluation of cccDNA-targeted therapies. Third, delivery and specificity remain major hurdles. Gene editing, epigenetic and metabolic agents face challenges of hepatocyte-specific delivery and off-target effects on host chromatin and physiology. Fourth, many strategies show context-dependent efficacy. The activity of many metabolic-epigenetic and immune-based strategies varies with disease stage, hepatic fibrosis, steatosis, and baseline immune function. Optimal cure regimens will likely require rational combinations that simultaneously silence cccDNA transcription, block new cccDNA formation, reduce antigenemia, and restore immune control.

## 8 Conclusion and future perspectives

This review integrates current evidence to illustrate how hepatocyte metabolic-epigenetic signaling serves as a master regulator of cccDNA reservoir activity, viral persistence, and immune tolerance in chronic HBV infection. Lifelong persistence of HBV is maintained by a highly orchestrated viral life cycle centered on the stable cccDNA minichromosome and its sophisticated interplay with host metabolic and epigenetic networks. From NTCP-mediated entry to metabolic-epigenetic cccDNA regulation and subviral particle-mediated immune evasion, HBV has evolved robust mechanisms to sustain its intrahepatic niche. Future efforts toward functional cure will require combinatorial regimens that disrupt cccDNA homeostasis via metabolic-epigenetic interventions and block tolerogenic antigen production while targeting viral entry and cell-to-cell transmission to prevent reservoir replenishment. Emerging strategies including nuclear receptor modulators, epigenetic agents, metabolic reprogramming, gene editing, capsid modulators, and transcript-targeted therapeutics have yielded encouraging preclinical and early clinical outcomes in suppressing viral activity. It is critical to emphasize that the vast majority of metabolic and epigenetic interventions targeting cccDNA remain confined to preclinical investigation, with only a handful of candidates progressing to early-phase clinical trials. Most importantly, transcriptional repression or partial reduction of cccDNA activity, which constitutes the primary outcome of most current metabolic-epigenetic strategies, is fundamentally insufficient to achieve a durable functional cure. These approaches may offer potential for achieving sustained HBsAg loss and durable cccDNA silencing when rationally combined, ultimately transforming the clinical management of chronic HBV infection.

Despite substantial advances delineating the metabolic-epigenetic control of cccDNA, several critical controversies and unresolved questions remain to be addressed. Most metabolic-epigenetic observations are derived from reductionist cellular systems; translation to the complex human liver microenvironment characterized by fibrosis, steatosis, and diverse immune cell infiltration remains a major translational bottleneck. Estimates of cccDNA turnover vary drastically across experimental systems, and precise measurement of cccDNA half-life in chronically infected human liver tissues continues to pose technical barriers. The *in vivo* contribution of hepatocyte cell-to-cell transmission also remains debated, as most supportive evidence originates from cell-culture models, and its quantitative impact on sustaining the intrahepatic reservoir in patients awaits further validation. Additionally, many metabolic intermediates and epigenetic modifiers exert context-dependent effects on cccDNA transcription. Most candidate pharmacological agents targeting this axis have only been evaluated in preclinical settings. Whether such strategies can achieve durable cccDNA silencing without unacceptable off-target hepatic toxicity requires long-term *in vivo* investigation.

Alongside unresolved mechanistic questions, clinical translation is also hampered by the lack of reliable tools to monitor intrahepatic cccDNA activity non-invasively. Clinically, multiple circulating surrogate biomarkers have been increasingly utilized to indirectly reflect intrahepatic cccDNA transcriptional activity, given the infeasibility of routine liver biopsy and direct cccDNA quantification. Quantitative HBsAg (qHBsAg) serves as the most commonly used serological marker reflecting viral transcriptional output (Ali et al., 2025), and a sustained decline in qHBsAg during antiviral therapy is generally interpreted as reduced transcriptional activity of intrahepatic cccDNA, although its direct correlation with intrahepatic cccDNA is not perfect (Chen et al., 2017). However, in patients with integrated HBV DNA, qHBsAg cannot reliably reflect true cccDNA activity, since integrated viral templates may constitute a major source of HBsAg particularly in HBeAg-negative patients and continuously produce HBsAg independent of episomal cccDNA transcription (Wooddell et al., 2017), and this same integration-derived antigen production further compromises the cccDNA specificity of HBsAg-based readouts. HBcrAg and circulating HBV RNA/pgRNA represent more specific surrogate markers for cccDNA-driven transcription. HBcrAg levels closely correlate with intrahepatic cccDNA abundance and transcriptional activity, and undetectable HBcrAg correlates with lower intrahepatic cccDNA and reduced transcriptional activity (Wooddell et al., 2017). Similarly, serum HBV RNA is mainly derived from cccDNA-originated pgRNA and acts as a real-time indicator of residual cccDNA transcriptional activity (Zhu et al., 2026), with proven value in predicting viral relapse after nucleos(t)ide analog discontinuation (Wu et al., 2024).

Nevertheless, these serum surrogates carry inherent limitations. None of these circulating biomarkers can precisely quantify the absolute size of the cccDNA reservoir or reliably discriminate transcriptionally silent from transcriptionally competent cccDNA, as a convenient and reproducible biomarker of cccDNA transcriptional activity remains lacking (Testoni et al., 2024). Under NA therapy, serum HBV DNA may drop rapidly below the detection limit even when transcription-competent cccDNA still resides in hepatocytes, and cccDNA remains detectable in the liver despite undetectable serum HBV DNA (Li et al., 2025). Moreover, once pgRNA reverse transcription is blocked, DNA-based serum readouts no longer truly reflect cccDNA activity, so such readouts may underestimate the residual reservoir activity (Huang et al., 2018). In addition, heterogeneous host immune responses and viral genetic variations can further decouple serum biomarker readouts from genuine intrahepatic cccDNA homeostasis (Huang et al., 2018). Emerging circulating molecular signatures, including cell-free HBV DNA fragments, exosomal HBV transcripts, and liver-derived epigenetic markers, have been proposed to improve the evaluation of cccDNA activity. However, the evidence for most of these approaches remains largely preliminary, and their clinical specificity, dynamic cutoff values, and long-term prognostic performance await validation. Collectively, the absence of a gold-standard non-invasive biomarker represents a major translational bottleneck for real-time assessment of cccDNA homeostasis, restricting rational design and timely efficacy evaluation of curative combination regimens.

Ultimately, resolving these open questions, particularly regarding the delivery specificity of gene editing approaches, the off-target effects of epigenetic modulators, and the clinical validation of non-invasive biomarkers, will be essential to facilitate the rational design of next-generation combination therapies and accomplish a durable functional cure for chronic HBV. Additionally, the impact of metabolic-epigenetic regulation on cccDNA may differ across clinical phases of chronic HBV infection, including immune tolerance, immune clearance, and the inactive carrier state, which remains an important dimension for future investigation.

## Glossary

HBV, hepatitis B virus; HCC, hepatocellular carcinoma; WHO, World Health Organization; HBsAg, hepatitis B surface antigen; NAs, nucleos(t)ide analogues; IFNs, interferons; Peg-IFN, pegylated interferon; cccDNA, covalently closed circular DNA; kb, kilobases; rcDNA, relaxed-circular DNA; Pol, polymerase; HBcAg, hepatitis B core antigen; HBeAg, hepatitis B e antigen; HBx, hepatitis B virus X protein; NTCP, sodium taurocholate co-transporting polypeptide; HSPGs, heparan sulfate proteoglycans; NPC, nuclear pore complex; pgRNA, pregenomic RNA; TDP2, tyrosyl-DNA phosphodiesterase 2; FEN1, flap endonuclease 1; LIG1, ligase 1; LIG3, ligase 3; dNTP, deoxyribonucleoside triphosphates; DDR, DNA damage response; ATR, ataxia telangiectasia and RAD3-related; TOP1, topoisomerase 1; TOP2, topoisomerase 2; preC, precore; BCP, basal core promoter; SVPs, subviral particles; HBc, hepatitis B core protein; PTMs, post-translational modifications; CP, core promoter; H3K4, histone H3 lysine 4; H3K27, histone H3 lysine 27; H3K9, histone H3 lysine 9; HDAC11, histone deacetylase 11; HAT1, histone acetyltransferase 1; H4K5, histone H4 lysine 5; H4K12, histone H4 lysine 12; SETDB1, SET domain bifurcated histone lysine methyltransferase 1; PRC2, polycomb repressive complex 2; PRMT5, protein arginine methyltransferase 5; PRMT1, protein arginine methyltransferase 1; H4R3, histone H4 arginine 3; H3K79, histone H3 lysine 79; KAT2A, lysine acetyltransferase 2A; NAD^+^, nicotinamide adenine dinucleotide; SIRT7, sirtuin 7; H3K122, histone H3 lysine 122; SUV39H1, suppressor of variegation 3-9 homolog 1; SETD2, SET domain containing 2, histone lysine methyltransferase; H3K36, histone H3 lysine 36; HDAC1, histone deacetylase 1; LLPS, liquid-liquid phase separation; FUS, fused in sarcoma; ABCF1, ATP binding cassette subfamily F member 1; SIRT1, sirtuin 1; CBP, CREB-binding protein; p300, E1A-binding protein p300; PCAF, p300/CBP-associated factor; SMC5, structural maintenance of chromosomes 5; SMC6, structural maintenance of chromosomes 6; HMGB1, high mobility group box 1; PRPF31, pre-mRNA processing factor 31; SUZ12, suppressor of zeste 12 homolog; SLF2, SMC5-SMC6 complex localization factor 2; PML, promyelocytic leukemia; HULC, highly upregulated in liver cancer; HAT1, histone acetyltransferase; CAF-1, chromatin assembly factor 1; HNF4α, hepatocyte nuclear factor 4α; RXR, retinoid X receptor; PPARα, peroxisome proliferator-activated receptor α; FXR, farnesoid X receptor; BA, bile acid; FXRα, farnesoid X receptor α; RXRα, retinoid X receptor α; BAs, bild acids; acetyl-CoA, acetyl coenzyme A; HATs, histone acetyltransferases; αKG, α-ketoglutarate; KDM4A, lysine demethylase 4A; H4K20, histone H4 lysine 20 Trimethylation; NK, natural killer; TNF-α, tumor necrosis factor-α; IFN-γ, interferon-γ; TRAIL, tumor necrosis factor-related apoptosis-inducing ligand; APOBEC3A, apolipoprotein B mRNA editing enzyme, catalytic polypeptide-like 3A; APOBEC3B, apolipoprotein B mRNA editing enzyme, catalytic polypeptide-like 3B; LTβR, lymphotoxin-β receptor; NF-κB, nuclear factor kappa B; ISG20, interferon-stimulated gene 20; IFN-α, interferon-α; STAT1, signal transducer and activator of transcription 1; STAT2, signal transducer and activator of transcription 2; DHBV, duck hepatitis B virus; MX2, MX dynamin-like GTPase 2; UBE2L3, ubiquitin-conjugating enzyme E2 L3; IL-6, interleukin-6; HNF1α, hepatocyte nuclear factor-1α; STAT3, signal transducer and activator of transcription 3; TGF-β, transforming growth factor-β; AID, activation-induced cytidine deaminase; UNG, uracil-DNA glycosylase; AP, apurinic/apyrimidinic; hnRNPA2B1, heterogeneous nuclear ribonucleoprotein A2B1; HSPB1, heat shock protein family B member 1; IMPα, importin-α; IMPβ, importin-β; WHV, woodchuck hepatitis B virus; HBcrAg, hepatitis B core-related antigen; AA, arachidonic acid; lncRNA, long non-coding RNA; HOXA-AS2, HOXA cluster antisense RNA 2; siRNA, small interfering RNA; MTA1, metastasis-associated protein 1; ACSS2, acyl-CoA synthetase short-chain family member 2; GlyT1, glycine transporter-1; mTORC1, mammalian target of rapamycin complex 1; CRISPR, clustered regularly interspaced short palindromic repeats; Cas, CRISPR-associated systems; LNP, lipid nanoparticle; ARCUS-POL, sequence-specific ARCUS meganuclease; VSV-G, vesicular stomatitis virus G protein; gRNA, guide RNA; HDV, hepatitis delta virus; CAMs, capsid assembly modulators; CAM-E, capsid assembly modulator-empty; CHB, chronic hepatitis B; NAPs, nucleic acid polymers; TDF, tenofovir disoproxil fumarate; ASOs, antisense oligonucleotides; GalNAc, N-acetylgalactosamine; MVA, modified vaccinia virus Ankara; TCR, T-cell receptor; HLA, human leukocyte antigen; qHBsAg, quantitative HBsAg.
